# Navigating and attributing uncertainty in future tropical cyclone risk estimates

**DOI:** 10.1126/sciadv.adn4607

**Published:** 2025-04-18

**Authors:** Simona Meiler, Chahan M. Kropf, Jamie W. McCaughey, Chia-Ying Lee, Suzana J. Camargo, Adam H. Sobel, Nadia Bloemendaal, Kerry Emanuel, David N. Bresch

**Affiliations:** ^1^ Institute for Environmental Decisions, ETH, Zurich, Switzerland.; ^2^ Federal Office of Meteorology and Climatology MeteoSwiss, Switzerland.; ^3^ Lamont-Doherty Earth Observatory, Columbia University, Palisades, NY, USA.; ^4^ Department of Applied Physics and Applied Mathematics, Columbia University, New York, NY, USA.; ^5^ Institute for Environmental Studies (IVM), Vrije Universiteit Amsterdam, Amsterdam, Netherlands.; ^6^ Royal Netherlands Meteorological Institute, De Bilt, Netherlands.; ^7^ Lorenz Center, Massachusetts Institute of Technology, Cambridge, MA, USA.

## Abstract

Future tropical cyclone risks will evolve with climate change and socioeconomic development, entailing substantial uncertainties. An uncertainty and sensitivity analysis of these risks is vital, yet the chosen model setup influences outcomes. This study investigates how much future tropical cyclone risks are driven by climate and socioeconomic changes, quantifies uncertainty from propagating alternate representations of these systems through the risk modeling chain, and evaluates how strongly each model input contributes to output uncertainty. By comparing these three elements—drivers, uncertainty, and sensitivity—across four distinct tropical cyclone models, we derive findings generalizable beyond individual model setups. We find that average tropical cyclone risk will increase 1 to 5% by 2050 globally, with maximum increases ranging from 10 to 400% by 2100, depending on tropical cyclone model choice, region, and risk model inputs, while the dominant source of uncertainty shifts with modeling choices. Last, we differentiate between aleatory, epistemic, and normative uncertainties, offering guidance to reduce them and inform better decision-making.

## Teaser

Quantifying, classifying, and comparing uncertainties in future tropical cyclone risks toward actionable climate decisions.

## INTRODUCTION

In recent years, catastrophe modeling has expanded beyond its traditional realm in the (re-)insurance industry to serve the broader global financial market and has also found increasing applications to humanitarian and sustainable development efforts. As climate change is represented in the models used, we refer to them broadly as climate risk models, a category that includes, but is broader than, catastrophe models that have a longer history in (re-)insurance. Many consultancies, financial technology firms, data providers, and investment advisory groups now offer information about localized physical climate risks, entering a technology arms race among climate services providers (*1*, *2*). However, the proprietary nature of their products inhibits both transparency and accessibility and makes it difficult to evaluate or compare them (*1*–*3*). Efforts to establish measurement and reporting standards are still evolving (*4*). Beyond insurance and finance, climate risk modeling is also being increasingly applied to inform adaptation decisions in development and humanitarian programs, where the potential for societal benefit is large (*5*, *6*). Here too, there is a pressing need for a better understanding of the quality and reliability of climate risk assessments (*7*, *8*).

Tropical cyclone (TC) risk provides a prime example of the challenges and complexities faced in the broader field of climate risk analysis. TCs are among the most destructive natural hazards, posing substantial threats to people (*9*) and assets (*10*) exposed to these events. In the future, TC risks are expected to increase further because of the warming climate and socioeconomic development (*9*, *11*, *12*). It is thus crucial to support at-risk communities with reliable and transparent TC risk assessments. However, providing reliable TC risk assessment is challenging because of uncertainties in the model input components and model structure (*13*). TC risks emerge from the interplay of TC hazards, the extent to which people and assets are exposed to these hazards, and the vulnerability of the exposed individuals and the (built) environment to these hazards (*14*). Climate risk models hinge on the interplay of these three components: hazard, exposure, and vulnerability (*15*). Each of these risk elements is subject to numerous uncertainties, and additional uncertainty emerges when they are combined. Meiler *et al.* (*16*) investigate uncertainties in the TC hazard model choice for present-day loss estimates. Assessing future TC risks requires additional modeling choices regarding the representation of future climate and socioeconomic systems. Each of those introduces its own uncertainties and is further confounded by the lack of verification data (*17*, *18*).

This study distinguishes three types of uncertainty—epistemic, aleatory, and normative—that are relevant to climate risk assessment, extending beyond the scope of TCs. Epistemic uncertainty arises from limited knowledge about the systems being modeled and involves the structural uncertainties in synthetic TC models, historical data quality, limitations in data availability, and understanding of environmental interactions (*19*). It includes scenario uncertainty, i.e., the unpredictability of future emissions scenarios (*20*–*23*), and model uncertainty (*20*–*22*), which here, for example, refers to the limitations of climate models, models used to generate synthetic TCs, or the exposure model to derive a spatially explicit map of asset values. Aleatory uncertainty stems from the inherent randomness of natural processes, such as climate variability that is internal or unforced by human influence (*19*). This type of uncertainty can be quantified through statistical methods, like Monte Carlo simulations, to estimate the probability distribution of outcomes. Normative uncertainty emerges from subjective decisions and ethical considerations in risk assessment processes, influencing the choice of valuation units and risk metrics (*24*–*26*). While not quantifiable like epistemic or aleatory uncertainties, normative uncertainty can be addressed through increased transparency, stakeholder engagement, and the integration of diverse ethical perspectives (*27*). The consequences of normative choices can also be quantified to the degree that alternate choices lead to alternate model inputs and modeling approaches. Such analyses of normative uncertainties, like the choice of output metric, may add a level of complexity, but they are possible and can provide valuable insights.

Given the complexities of epistemic, aleatory, and normative uncertainties, a systematic approach to uncertainty quantification in climate risk assessment emerges as a critical need. In this study, we use the open-source, peer-reviewed, probabilistic climate risk modeling platform CLIMADA (*15*) to compute future TC risk estimates and quantify and attribute associated uncertainties. To achieve this, we use the uncertainty and sensitivity quantification (unsequa) module (*13*), a tool already integrated within CLIMADA, enabling comprehensive uncertainty and sensitivity analyses for all CLIMADA-based risk calculations. Different methods for uncertainty and sensitivity analysis have been proposed in the scientific literature (*13*, *17*, *28*, *29*). Typically, uncertainty analysis quantifies the overall uncertainty, while sensitivity analysis identifies how different input factors contribute to this uncertainty (*28*). Unlike commonly used local sensitivity methods that vary each input one at a time, our global sensitivity analysis varies input factors all at a time across their entire ranges (*17*). A key benefit of global sensitivity analysis is its ability to simultaneously vary all inputs, enabling us to attribute their relative contributions to overall uncertainty and understand input factor interactions. Given this function, we use the terms uncertainty attribution and sensitivity analysis interchangeably.

We systematically quantify uncertainties and sensitivities in future TC risk change estimates in the middle and at the end of the century, encompassing uncertainties in all risk model components: hazard, exposure, and vulnerability. In other words, we approach this analysis from a climate risk modeler’s perspective, incorporating uncertainties in all essential risk assessment components and reflecting model choices any risk modeler is confronted with during such assessments. Linking open-source climate risk models like CLIMADA with uncertainty and sensitivity assessments is crucial for reducing or, at least, understanding these uncertainties (*30*, *31*). Our perspective thus differs in scope and focus from other climate-related studies that solely address the physical aspects of climate change without considering socioeconomic factors.

Contrasting results from two previous studies assessing uncertainties and sensitivities in the quantification of future TC risks, each using a different TC hazard model, show that the results of such uncertainty and sensitivity quantification depend on the scope of the study, which is defined a priori by investigator choice—In other words, uncertainty assessment is itself uncertain (*17*, *30*, *32*). This type of uncertainty is increasingly recognized as a substantial issue in uncertainty analysis auditing [e.g., (*33*, *34*)]. To address this issue, we perform a comprehensive comparison of uncertainty and sensitivity analyses across four distinct TC hazard models and risk model setups. As some scenarios are implemented in some models but not others, our comparison is not of all possible combinations in principle but of all combinations of these presently available to the risk modeler. This comparison allows us to evaluate and understand how uncertainties in model inputs, structures, and assumptions affect the outcomes of the models. In addition, it enables us to derive generalizable implications beyond the scope of individual model setups, which is not achievable with a single model structure or setup alone.

This study operates on two distinct levels of investigation, providing a comprehensive analysis of uncertainties in future TC risk estimation. First, we investigate the uncertainty and sensitivity in estimated future TC risk that arises from a risk modeler’s choice of alternative representations of hazard, exposure, and vulnerability components. Second, we repeat this approach over four different, global-scale, academic models, differing in structure and approach, to generate different future TC event sets. These models are used to downscale multiple emission scenarios and global climate models (GCMs) for two future periods. Specifically, we contrast TC event sets from two statistical-dynamical TC models, the Massachusetts Institute of Technology (MIT) model (*35*, *36*) and Columbia HAZard model (CHAZ) (*37*, *38*), the fully statistical model STORM (*39*, *40*) and a more simplistic, statistical model (IBTrACS_p) applying a random walk algorithm (*12*, *41*) to historical TC observations from the International Best Track Archive for Climate Stewardship (IBTrACS) (*42*). In addition, we vary other factors in the hazard component that span all four TC models, such as the choice of parametric wind field or event subsampling.

Across all four models, we vary the same factors in the exposure and vulnerability components. In particular, we use economic growth factors from all five shared socioeconomic pathways (SSPs) (*43*) to approximate and analyze socioeconomic development, thereby addressing growth uncertainties in future asset value exposure. These SSP scenarios, which describe diverse future societal trajectories, are informed by gross domestic product (GDP) projections from three distinct research institutions [Organization for Economic Cooperation and Development (OECD) (*44*), International Institute for Applied Systems Analysis (IIASA) (*45*), and Potsdam Institute for Climate Impact Research (PIK) (*46*)], each providing alternative interpretations of economic development under the SSP framework. We do not speculate on future changes in the vulnerability function due to the current knowledge gap in this matter. Instead, we explore uncertainties by adjusting the slope parameter of regionally calibrated vulnerability functions based on historical data (*47*) within a wide range. Given the limited availability of openly available impact functions, our approach relies on functional shapes based on established work, such as Emanuel (*48*), which are typical in TC risk assessments for direct economic impacts. Different fields interpret the vulnerability concept differently, which can influence the choice of functional shapes used (*49*).

**Fig. 1.**
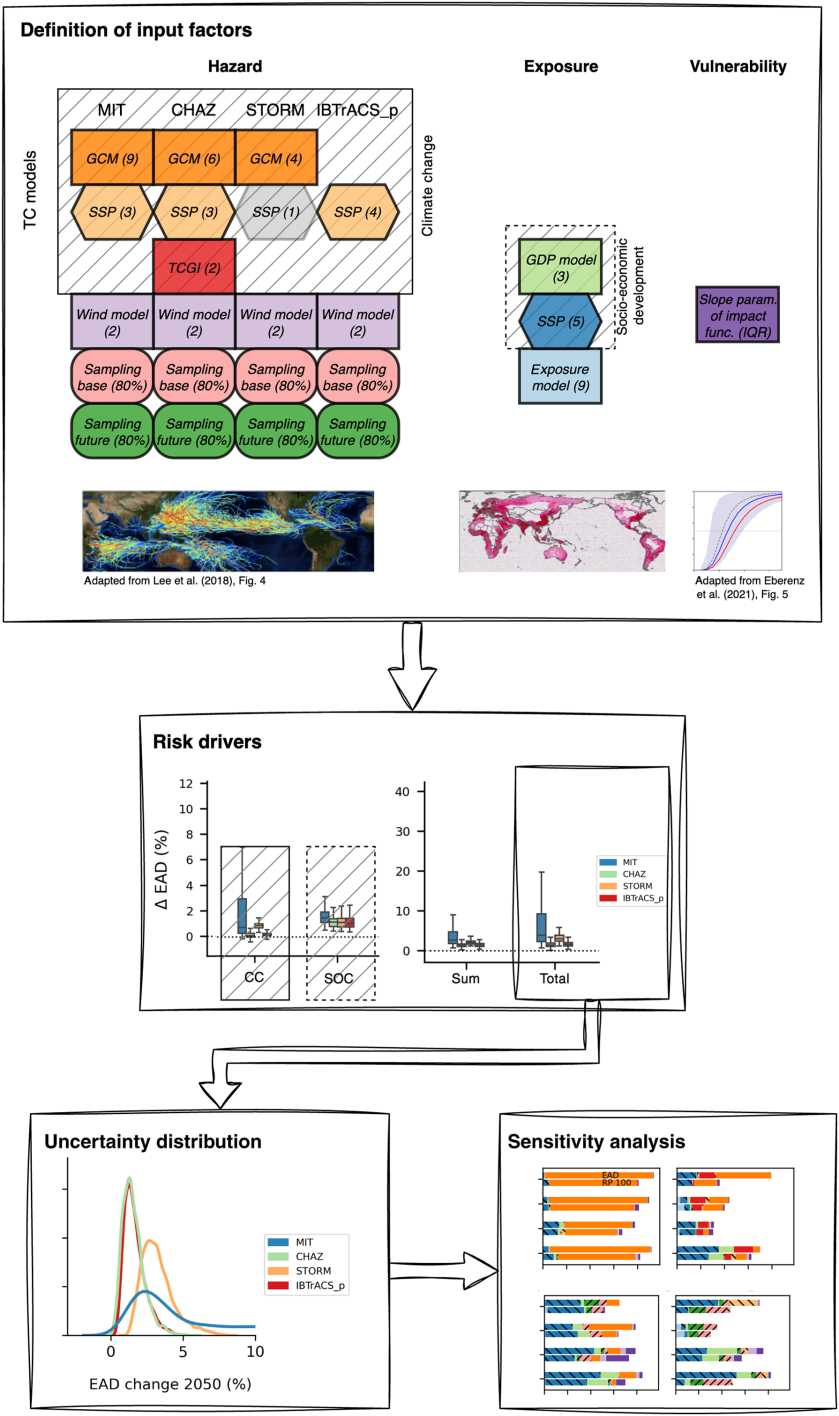
Schematic overview of uncertainty and sensitivity analysis. The definition of input factors shows which risk model components are varied (top box). The characterization of their variability space is detailed in Uncertainty and sensitivity analysis section in Materials and Methods. Input factors related to the hazard are defined separately for each TC model (MIT, CHAZ, STORM, and IBTrACS_p). TC model–related input factors (TC models section in Materials and Methods) are labeled as such. Climate change modulates the hazard, socioeconomic development exposure; input factors affected by either are hatched. Input factors representing primarily aleatory uncertainty are shown in rounded boxes; epistemic uncertainty in square boxes, of which rectangular boxes represent model uncertainty and hexagons scenario uncertainty. The information in brackets indicates the parameter range (discrete or continuous) from which the input factors are sampled. Note that STORM is run only for SSP5-8.5, so SSP is present in the STORM model structure but not sampled in the analysis. The respective box is gray and marked (*1*). An illustration at the bottom of the box depicts the three risk model components. The risk calculation is repeated (>20000) times for all combinations of input factors. We first study risk drivers (middle box, Drivers of future TC risk change section) and then the uncertainty distribution for each TC model setup (lower left box, Uncertainty of future TC risk change section). Lastly, sensitivity indices are calculated from these distributions (lower right box, Sensitivity of future TC risk change section).

All these alternative representations of hazard, exposure, and vulnerability, including the choice of different TC models, reflect typical risk modeling choices and the current availability of globally consistent, open-access risk model input data. We do not claim that this range of available input data and modeling setups represents the range of uncertainty of the underlying modeled systems; hence, in the analysis that follows, our attribution of uncertainty to specific aspects (hazard, exposure, and vulnerability) indicates those aspects as represented in typical risk modeling choices. We perform the uncertainty and sensitivity analysis for future TC risk change estimates based on all possible combinations of input factors, relying on a numerical Quasi-Monte Carlo scheme (*50*) to repeat the risk calculation many times (>20,000). Specifically, we assess the key drivers (Drivers of future TC risk change section), quantify uncertainties (Uncertainty of future tropical cyclone risk change section), and analyze sensitivities (Sensitivity of future tropical cyclone risk change section) in future TC risk estimates. A schematic overview of the uncertainty and sensitivity analysis conducted in this study is shown in Fig. 1.

While previous studies have examined how variations in hazard, exposure, and vulnerability translate into risk, this study includes four distinct hazard models (and their variations) alongside different representations of exposure and vulnerability in the assessment of future TC risks and associated uncertainties. Consequently, it provides valuable insights into the structural differences between TC models, their implications for risk assessment, and the interpretation of uncertainty and sensitivity analyses of future TC risks. We synthesize aspects of model choice, model complexity, and their implications for model development and decision-making.

## RESULTS

### Drivers of future TC risk change

Future TC risks change because of both the warming climate and socioeconomic development. Here, we first evaluate the individual contributions of these two key drivers to future TC risk estimates. To consider the influence of climate change on risk, we hold exposure constant at a reference state while using varying future climate hazard representations. Conversely, to assess the impact of socioeconomic factors, we keep the hazard data fixed at the present-day baseline, allowing socioeconomic conditions, i.e., the exposure layer, to vary. Then, we study future TC risk change estimates of both key drivers acting together. We conduct and contrast this analysis for the four TC models. TC risks are expressed by the common metric of expected annual damage (EAD) and 100-year damage event (100-year event in short), reported as relative changes (in %) compared to present-day baselines. The EAD is the integrated value of impacts across all probabilities and exposure points [cf. equation 5 in Aznar-Siguan and Bresch (*15*)] and is commonly used as a proxy for risk-based insurance premiums in catastrophe modeling (*51*). The 100-year event is an extreme event expected to occur once every 100 years on average, which translates to a 1% chance of occurring in any given year. We simulate direct economic damage in the form of impact on the built environment from a given TC hazard set. For this, we use a spatially explicit map of asset values as exposure (see section Asset exposure representations in Materials and Methods) and a regionally calibrated set of impact functions (*47*). Note that we only consider wind as the driving physical hazard for the resulting socioeconomic impact. However, the regionalized impact functions used in this study (*47*) implicitly capture damages from storm surges and rainfall-induced freshwater flooding because they were calibrated to total damage values. We present results for four study regions: North Atlantic/Eastern Pacific, North Indian Ocean, Southern Hemisphere, and North Western Pacific (see section Study regions in Materials and Methods). We limit the results’ description to the EAD in this section because the corresponding key findings for the 100-year event are comparable (cf. figs. S1 and S3).

**Fig. 2.**
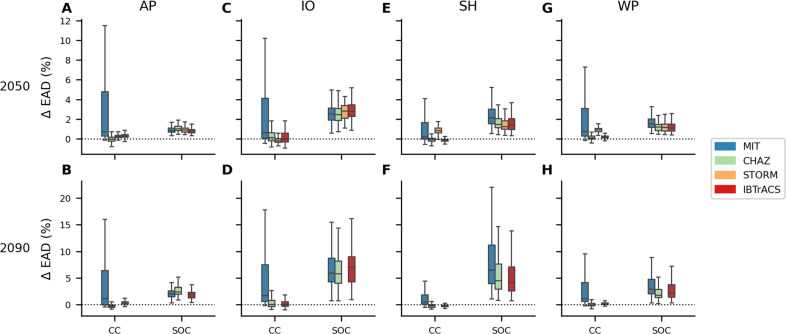
Drivers of future TC risk change. Relative change in EAD by 2050 (**A**, **C**, **E**, **G**) and 2090 (**B**, **D**, **F**, **H**) due to climate change (CC) and socioeconomic development (SOC) with respect to the historical baseline. The relative change EAD is reported for the four study regions [North Atlantic/Eastern Pacific (AP) (A) (B), North Indian Ocean (IO) (C) (D), Southern Hemisphere (SH) (E) (F), and North Western Pacific (WP) (G) (H)]. Boxplots are shown for the four models MIT (blue), CHAZ (green), STORM (orange), IBTrACS_p (red), and display the interquartile range (IQR) for the uncertainty over all input factors (see Materials and Methods), while the whiskers extend to 1.5 times the IQR. More extreme points (outliers) are not shown. Note that STORM results are only available for 2050.

Climate change generally affects the median TC risk changes comparably across hazard models, study regions, and periods (Fig. 2, left boxplots in all panels). Specifically, the median change in EAD is usually on the order of 0 to −1%. However, the uncertainty in TC risk change estimates is notably higher for all MIT hazard results than the other hazard model outputs, as can be derived from the width of the interquartile range of the boxplots shown in Fig. 2. Furthermore, maximum values for climate change–driven EAD increase from the MIT hazard reach 20% (45%), 19% (28%), 6% (8%), and 14% (14%) in the North Atlantic/Eastern Pacific, North Indian Ocean, Southern Hemisphere, and Western Pacific in the middle (at the end) of the century (table S6). In contrast, maximum risk increases from the other hazard models do not exceed the 5% mark except in the North Indian Ocean. There, climate change raises EAD values from CHAZ by 10% (9%) and IBTrACS_p by 23% (23%) in 2050 (2090), respectively. Only the results from STORM remain low because of known high-intensity biases in the reference period hazard set (*16*, *32*). The North Indian Ocean is, furthermore, the region where uncertainties in climate-driven risk change are highest across all hazard models. In addition, median TC risk changes are lowest in the Southern Hemisphere over all regions, including negative values for CHAZ and IBTrACS_p. In other words, climate-driven TC risk decreases in these cases. Indeed, we find negative minima of ~ 0 to −1% for all hazard models and regions (table S6). The effects of climate change on key variables important for TC risks, like intensity and frequency, are complex (*38*, *52*), yielding a suite of results from slight risk decreases to risk increases, and trends modeled by mid-century generally become more pronounced further into the future.

Socioeconomic development emerges as the predominant driver for TC risk increase, as can be seen from the greater increase in risk associated with socioeconomic development alone than with climate change alone (Fig. 2, right boxplots in all panels). This is consistent across all hazard models, using the same future socioeconomic representation for each. Notably, any difference between the hazard models stems primarily from their distinct present-day baseline. Specifically, the median EAD changes driven by socioeconomic development are around 1 to 2% by 2050. In regions like the North Atlantic/Eastern Pacific and Western Pacific, this is roughly double the changes attributed to climate change. However, in the North Indian Ocean, median values are higher: 2.5 to 3% [and 6 to 7% by 2050 (2090)], which is about four times the climate change contributions. Furthermore, the uncertainty tied to socioeconomic development is more pronounced in the Southern Hemisphere compared to the other three regions. Last, when considering the hazard sets CHAZ, STORM, and IBTrACS_p, socio-economic development presents more uncertainty than climate change. In contrast, for MIT-based calculations, climate change is the more uncertain risk driver.

Next, we assess the total TC risk increase, factoring in both climate change and socioeconomic development. Notably, the total TC risk increase, as depicted in fig. S2 (total; right boxplots in all panels), are not simple sums or products of risk increases attributed only to climate change or only to socioeconomic development, suggesting some further interdependencies between these drivers [fig. S2 (sum; left boxplots in all panels)].

Median EAD raises by 0.9% (CHAZ) to 2.3% (MIT), 2.1% (STORM) to 5.3% (MIT), 1.1% (IBTrACS_p) to 3.8% (MIT), and 1.4% (CHAZ) to 3.8% (MIT) in the North Atlantic/Eastern Pacific, North Indian Ocean, Southern Hemisphere, and Western Pacific by 2050. In all regions, the median risk increase is highest for the MIT hazard, while the other three models tend to cluster around similar values, with STORM producing slightly higher results in the Southern Hemisphere and Western Pacific than CHAZ and IBTrACS_p. By the end of the century, the median risk increases further, reaching levels approximately two to three times the increase in EAD estimated for 2050. Furthermore, maximum total EAD increases by 2090 span from 11% (CHAZ) to 264% (MIT), 134% (CHAZ) to 393% (MIT), 22% (IBTrACS_p) to 159% (MIT), and 15% (CHAZ) to 96% (MIT) in the North Atlantic/Eastern Pacific, North Indian Ocean, Southern Hemisphere, and Western Pacific respectively, highlighting the notable uncertainty in these results (table S6). We focus on total risk increases for the remainder of the study as described in this last paragraph.

### Uncertainty of future TC risk change

**Fig. 3.**
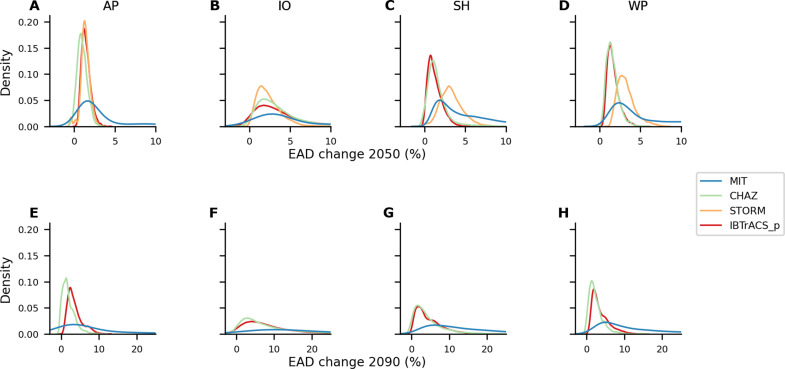
Uncertainty distribution of TC risk change. Kernel density estimation plots showcasing the uncertainty distribution of estimated relative change in EAD across study regions AP (**A**) and (**E**), IO (**B**) and (**F**), SH (**C**) and (**G**), and WP (**D**) and (**H**) for the years 2050 (A), (B), (C), and (D), and 2090 (E), (F), (G), and (H). Each subplot represents a specific region and year combination, with different models (MIT, CHAZ, STORM, and IBTrACS_p) depicted in distinct colors. Note that the model STORM only provides data for 2050. Each plot shows a normalized probability distribution with an integral sum of 1. The *x* axis is truncated in some figures, potentially influencing the interpretation of distribution tails, particularly for the MIT hazard–based results.

To quantify uncertainty in future TC risk change estimates, we calculate a probability distribution of outcomes across all combinations of risk model input factors. These factors represent variations in future climate and socioeconomic systems, including climate change projections, socioeconomic pathways, and alternative vulnerability functions. The resulting probability density plots represent the distribution of model outputs across the range of plausible input factor combinations, providing insight into the variability and relative frequency of outcomes rather than the likelihood of real-world events or scenarios. We repeat this analysis for four distinct TC hazard models, each with slightly different input factors (Fig. 3). In this section, uncertainty refers to the range of outcomes resulting from all possible combinations of input factors. We evaluate and contrast these uncertainties across the four TC models. We present the main findings for uncertainties of future TC risk change, focusing on changes in EAD, consistent with the preceding section. For results of the 100-year event, which are comparable, see fig. S4.

Figure 3 presents the probability density distributions of the total TC risk change, derived from the same data as the boxplots (total) in fig. S2. We identify density peaks of EAD change for CHAZ, STORM, and IBTrACS_p hazard sets in each region and both future periods around 1 to 3%. The density distributions from the MIT model, however, peak at higher values, consistent with the assessment of median total TC risk change from the previous section. When considering both risk metrics—EAD (Fig. 3) and the 100-year event (fig. S4)—we observe that their density distributions peak at very similar values for each combination of region, year, and hazard model (table S1). The consistency in peaks of density distributions for EAD and 100-year event aligns with our finding that socioeconomic development is the predominant driver for total TC risk change (Fig. 2). Socioeconomic development affects all events uniformly by changing exposure across the entire probability distribution, explaining the similar peak locations in both metrics. If climate change were the dominant driver, we would expect divergent patterns between EAD and 100-year event peaks, reflecting its potentially differential impact on frequent versus rare events; the observed similarity instead points to the uniform influence of socioeconomic development across all event probabilities.

Conversely, when examining the entire probability density distribution, the MIT results display a notably broader distribution compared to the other three hazard sets, a finding consistent with results from Fig. 2. The width of a distribution can serve as an indication of its associated uncertainty. Drawing from insights in the previous section, the width of the MIT-based distribution can be interpreted as an imprint of the uncertainties associated with climate change as a more uncertain risk driver. In contrast, the similar shapes of distributions from CHAZ, STORM, and IBTrACS_p models indicate socioeconomic development as their main source of uncertainty, as corroborated by Fig. 2. Furthermore, we observe wider distributions for results in 2090 compared to 2050 for all hazard models, related to increasing uncertainty in time. While this analysis provides insights into the overarching uncertainty, a more detailed examination of individual input factors is essential. In the following section, we explore these factors in detail through a sensitivity analysis.

### Sensitivity of future TC risk change

Sensitivity analysis helps identify and quantify the relative importance of individual input factors for the output uncertainty of future TC risk change estimates described in the last section. The model input factors and their parameter ranges are defined to capture the inherent uncertainties in the different components related to the representation of future TC hazards, exposure, and vulnerability. This sensitivity analysis complements our uncertainty quantification by providing insight into which input factors contribute most notably to the overall uncertainty in our TC risk change estimates. By performing this analysis across our four TC models, we can also assess how the importance of different factors varies between models. Here, we present first-order and total-order Sobol sensitivity indices (*53*, *54*) to assess the impact of the input factors on our TC risk change calculations. First-order sensitivity indices measure the effect of variations in a single input factor. They are often used to rank the input factors according to their relative contribution to the output variability (ranking). Total-order indices evaluate the cumulative effect, considering all factors and their potential interactions. They are commonly used for screening, aiming to identify the input factors—if any—with negligible influence on the output variability (*28*). We note that not all hazard models encompass all input factors (section Uncertainty and sensitivity analysis in Materials and Methods).

**Fig. 4.**
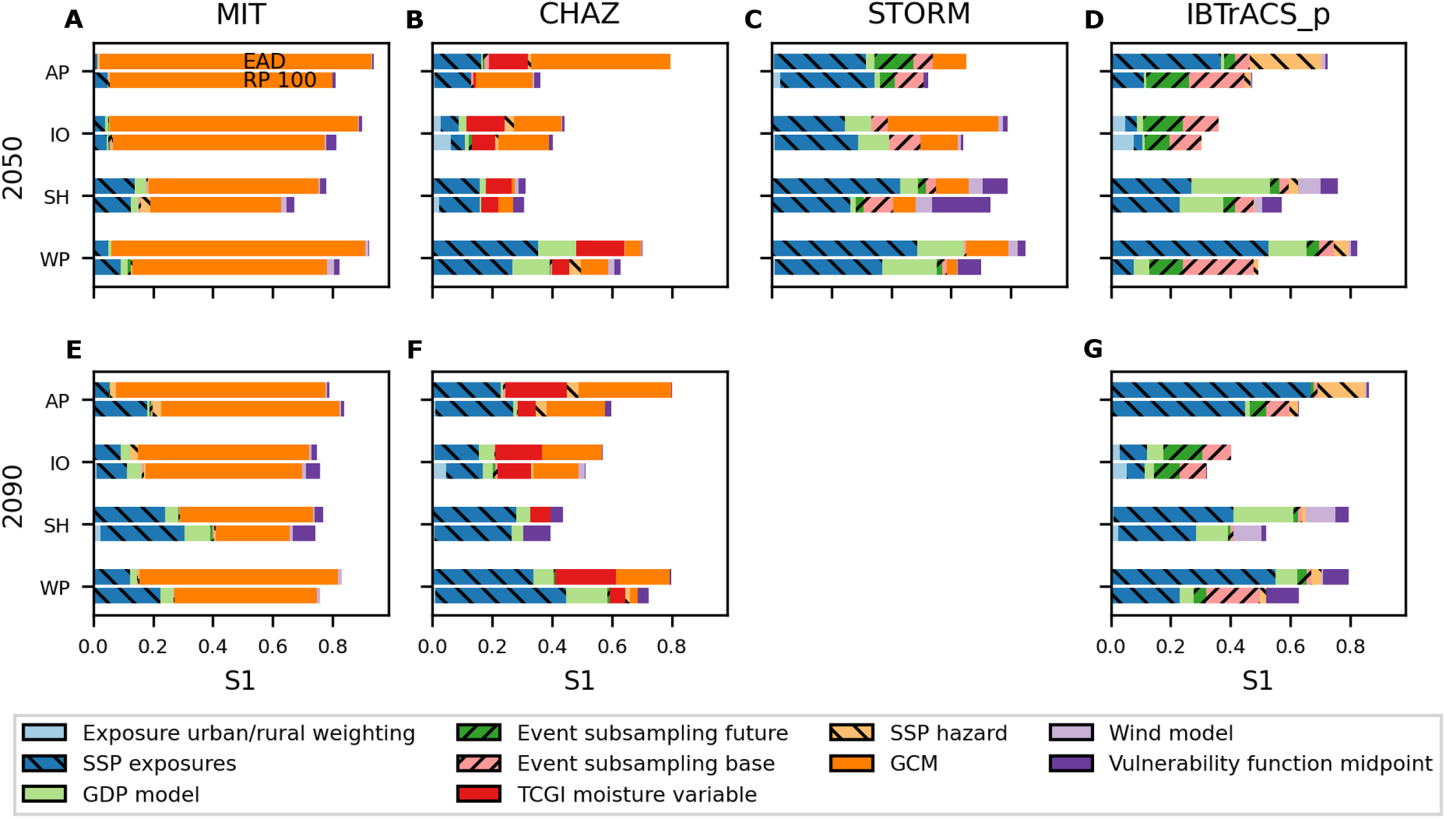
Sensitivity indices of future TC risk change. First-order Sobol sensitivity indices for future [2050 (**A**, **B**, **C**, **D**) and 2090 (**E**, **F**, **G**)] TC risk change calculated with the four models MIT [(A) and (E)], CHAZ [(B) and (F)], STORM (C), and IBTrACS_p [(D) and (G)], expressed as % change in EAD (upper bar for each hazard model, time, and region) and 100-year event values (RP 100, lower bar for each hazard model, time, and region) over the four study regions (AP, IO, SH, and WP) and all input factors (different colors). Input factors that primarily constitute aleatory uncertainty are shown in forward-slanting hatching; scenario uncertainty in backward-slanting hatching. Vulnerability function midpoint describes the impact function; wind model, GCM, SSP hazard, TCGI moisture variable, event subsampling base, and event subsampling future pertain to the hazard component; GDP model, SSP exposure, and exposure urban/rural weighting relate to the exposure. Note that STORM results are only available for 2050. Note that certain input factors apply to only one or a subset of models, c.f. Table 1 and Fig. 1.

The highest sensitivity indices describe the dominant source of uncertainty for future TC risk changes, which varies between the different hazard models. In the MIT model–based analyses, the highest sensitivity index stems from the choice of GCM used in downscaling TC events sets (GCM) (Fig. 4, A and E). Conversely, the SSP-based scaling of the exposure points (SSP exposure) generally exhibits the largest sensitivity for all other hazard models. Specifically, this holds for most results in the Southern Hemisphere and Western Pacific for the CHAZ, STORM, and IBTrACS_p and both future periods. In the North Indian Ocean, sensitivity indices are highest for input factors related to the hazard component [GCM, TC genesis index (TCGI) moisture variable, and event subsampling base/future], and results in the North Atlantic/Eastern Pacific follow no consistent trend beyond the primary observations mentioned. A detailed compilation of the most important sensitivity indices for future TC risk estimates can be found in table S2.

Moreover, the sensitivity analysis reveals several distinctive patterns. First, the GCM choice (GCM) is more important in the North Atlantic/Eastern Pacific, North Indian Ocean, and Western Pacific than in the Southern Hemisphere for the three hazard models (MIT, CHAZ, and STORM), which encompass this input factor. This pattern largely aligns with regions where uncertainties in climate change as a risk driver exceed uncertainties from socioeconomic development (see Fig. 2). Furthermore, the GCM choice is more important for changes in EAD than in the 100-year event. Second, for CHAZ model–based sensitivity analyses, the moisture variable within the TCGI is mostly of equal importance for the TC risk change uncertainty as the GCM choice (GCM) (Fig. 4, B and F). Third, the variability in event subsampling for baseline and future hazard sets (Event subsampling base/future) is most pronounced in the IBTrACS_p–related result (Fig. 4, B and G), in contrast to the other hazard models.

Next, we evaluate the total-order sensitivity indices (total effects) across the four hazard models. Namely, total effects are notably increased for CHAZ hazard–based results compared to their first-order indices, meaning that this model setup encompasses many interactions between input factors (fig. S5). In contrast, total-order sensitivity indices broadly mirror the ranking and distribution of the first-order indices for MIT- and STORM-related results. Moreover, in the IBTrACS_p–based sensitivity analysis, total effects include influences from the wind model choice (wind model), a nearly irrelevant factor in all other hazard sets.

Last, we emphasize that sensitivity analysis is always specific to the choice of risk metric. To illustrate this, we show the implications of assessing TC risk in absolute terms versus changes relative to a baseline. For absolute TC risk estimates, the primary source of uncertainty across all hazard models is the input factor associated with the vulnerability function (Vulnerability function midpoint), as depicted in figs. S6 and S7 and first discussed by Meiler *et al.* (*30*). Thus while the choice of vulnerability function is highly influential on the total risk that we calculate, this influence is much less apparent when we compare changes in risk calculated with the same vulnerability function. The vulnerability function is a critical link between hazard intensity and exposure in our modeling setup representing the relative degree of damage for any given wind speed. In assessments of absolute risk, it serves as an actual representation of this aspect of vulnerability. However, in assessments of relative risk change, it is fixed and acts as a mere translation tool, converting hazard intensities to risk changes.

## DISCUSSION

Our results on the key TC risk change drivers show that while both climate change and socioeconomic development influence TC risk changes, socioeconomic factors are the predominant drivers of median increased risk across all hazard models. While studying these drivers in isolation provided distinct insights, their combined effects reveal nontrivial interactions. This suggests that simply summing or multiplying their individual effects may not fully capture the complexity of their combined impact on TC risk or the nuances in the uncertainty and sensitivity analysis of these risk estimates. It underscores the importance of integrating both drivers from the onset in risk assessments to ensure a comprehensive understanding.

We report that median TC risk increases 1 to 5% by 2050 across all models and global study regions, perhaps a small enough change to be considered indistinguishable from zero for some purposes. However, the estimated maximum risk increases by the end of the century range from 10 to 400% depending on the hazard model choice and region. To the extent that we cannot rule out either any particular hazard model or any socioeconomic scenario, this suggests a much less optimistic view, with the potential for large increases in risk. Furthermore, we consider TC wind hazard only and do not explicitly include the potentially compounding effects of growing TC rainfall rates, storm surge heights, and sea level rise (*52*). While some water-related impacts are indirectly captured through the vulnerability functions, these factors likely exacerbate future TC risk increases further.

### Hazard model–specific findings

Comparing the key risk drivers across hazard models (Drivers of future TC risk change section), we find that for TC risk estimates based on the MIT model, climate change emerges as a more significant source of uncertainty than socioeconomic development. In contrast, for STORM, CHAZ, or IBTrACS_p–based results, socioeconomic development dominates the uncertainty (Fig. 2). Turning to the sensitivity analysis (Sensitivity of future TC risk change section), we observe that for results from the MIT model, the choice of GCM contributes most to the output uncertainty (Fig. 4), while for risk change estimates using the other three hazard models, the SSP-based exposure scaling (SSP exposure) has the highest sensitivity index. These findings highlight how the structural differences among the hazard models lead to varying sensitivities and responses to climate and socioeconomic drivers, shaping their respective contributions to uncertainty in the risk estimates. For TC risk estimates based on the MIT model, climate change is the more uncertain risk driver than socioeconomic development (Fig. 2), and the choice of GCM dominates the output uncertainty (Fig. 4). Conversely, when using STORM, CHAZ, or IBTrACS_p, socioeconomic development is the more uncertain risk driver than climate change, and the SSP-based exposure scaling (SSP exposure) has the highest sensitivity index. This difference is particularly notable when contrasting results from the two statistical-dynamical TC hazard models CHAZ and MIT. In a previous study solely based on MIT TC hazards, we found a positive relationship between the climate sensitivity of GCMs used to downscale TCs and the corresponding increase in TC risk (*30*). This increase is linked to the scaling of TC potential intensity with global warming (*55*), which in turn is a strong predictor for TC genesis potential indices (*56*–*58*). These indices again influence TC hazard frequencies and intensities, which are critical characteristics for TC risk assessment. Given the similar TC modeling approach to the MIT model, we expected to find a comparable relationship in the CHAZ-based results. Both models are statistical-dynamical models that downscale TCs from GCMs or reanalysis data and rely on three components: genesis, track, intensity. Although the MIT and CHAZ TC models differ in their specific genesis and intensity model, they are more similar to each other than to the purely statistical models STORM or IBTrACS_p. Unexpectedly, we found no notable relationship between transient climate response (table S5) as a measure of climate sensitivity and changes in CHAZ-based TC risk estimates (figs. S8 and S9) and CHAZ frequency (fig. S10) and intensity changes (fig. S11).

Building on this, we assert that the MIT model’s greater sensitivity to climate change should not be interpreted as increased uncertainty in the negative sense. The observed difference in sensitivity stems from the differing model structures. The MIT model’s intensity component solves dynamic equations, while the other models’ intensity components are statistical, with CHAZ using a statistical intensity model based on physical parameters. These differences suggest varying sensitivities to climate change across the models. We emphasize that our results do not attribute superiority or inferiority to any model. Rather, they reflect the current state of the field, where multiple approaches to TC modeling coexist. Since these responses of TCs to climate change are indeed uncertain (*52*)—with the response of TC frequency uncertain even in sign (*59*)—this uncertainty in our results may not be reducible given present science. It is not obvious, given the small size of our multimodel ensemble, that the real uncertainty might not be even larger, i.e., that some possible TC hazard models might show changes with climate either larger or smaller than those in our ensemble.

### Implications for interpretation of results, model development, and decision-making

In a previous study, we interpreted the importance of the GCM choice for MIT-based TC risk change estimates as an indication of the relatively advanced state of modeling of TC hazard, and a consequence of the greater complexity of the MIT model, compared to the exposure and vulnerability models (*30*). However, our current findings suggest a more nuanced narrative about the relationship between model complexity and uncertainty in TC risk estimation. Although CHAZ incorporates an additional hazard-related input factor (TCGI moisture variable; a detailed discussion of the role of TGCI for TC risk change estimates is provided in the Supplementary Materials) compared to the MIT model, its range of outputs in our risk estimation framework is narrower. This finding aligns with Puy *et al.*’s (*60*) suggestion that increased model complexity does not inevitably lead to higher uncertainty. In contrast, the influence of additional parameters on overall uncertainty depends on their specific roles and interactions within the model structure.

In our specific case, we observed that the relative magnitude and uncertainty associated with each input component of the risk model also play a crucial role in interpreting uncertainty analysis results. Specifically, we found that socioeconomic development is the dominant risk driver in the STORM, CHAZ, and IBTrACS_p models, while the MIT model shows climate change as equally important (Fig. 2). This results in a narrower uncertainty distribution in TC risk change estimates from STORM, CHAZ, and IBTrACS_p compared to the MIT model (Fig. 3). Therefore, we hypothesize that in cases where socioeconomic development is the notably stronger risk driver, the uncertainty in the climate change components diminishes in relevance simply because of the substantial impact of socioeconomic development. Understanding the magnitude and relative roles of these risk drivers is thus essential for interpreting the results of the uncertainty analysis and for reflecting on model complexities and effective dimensions, providing valuable insights for further model development.

While the mathematical concepts are straightforward—where magnitude often corresponds to the mode (peak) of probability density distributions and uncertainty affects the distribution’s width (spread or variance) (*13*, *17*, *28*)—grasping their practical implications is important. For risk analysts and decision-makers, the balance between considering the full range of possible outcomes, including allegedly improbable tails, and focusing on the peaks of distributions hinges on their level of risk aversion and the stakes involved. Low-risk aversion allows for prioritizing the most probable outcomes, streamlining decision-making toward the dominant risk drivers (magnitude), while uncertainties become secondary. For instance, an insurance underwriter may decide to focus on the central tendencies of the TC model output in a low-activity TC season for a low-stakes underwriting decision. In contrast, high-risk aversion necessitates a comprehensive analysis of all eventualities, in which case the significance of the central peak diminishes relative to uncertainty. A nuclear power plant operator, for example, needs to implement extensive safety measures against low-likelihood, high-impact TCs, prioritizing protection. Such a decision-maker would likely consider the upper bounds of our uncertainty ranges across all models to account for worst-case scenarios. This nuanced approach enables tailored risk management strategies that align with both the decision-maker’s level of cautiousness and the specific context of the decision (*61*).

These conclusions extend beyond the specific context of TC risks and are relevant for many types of hazards and climate risk information in general. Our study provides valuable insights for risk modelers, model developers, and global stakeholders such as the (re-)insurance industry and humanitarian organizations, which often require aggregated climate risk outputs to inform broad-scale strategies (*62*, *63*). Our comprehensive and structured uncertainty assessment enhances the transparency and richness of climate risk information, offering a blueprint for creating more actionable outputs for these applications. While this study does not approach many uncertainties related to how risk plays out differently in diverse local contexts, it can help inform such local studies by making uncertainties in larger-scale modeling more transparent.

### Classification of uncertainties and their implications

The outcomes of our uncertainty and sensitivity analyses reveal a strong dependency on the chosen risk model components, underscoring the necessity for careful interpretation and caution when extrapolating findings beyond the model boundaries. Our findings demonstrate not only that uncertainties vary with the TC model used but also that the relative sensitivities to different input factors shift as well. By mapping these outcomes to the aleatory, epistemic, and normative types of uncertainty considering their quantifiability and potential for reduction, we aim to illustrate how these analyses can be extended to generate actionable insights that extend beyond the immediate model setup. While the results presented in this study provide a comprehensive framework for understanding the combined effects of multiple sources of uncertainty, additional processing and tailored visualizations are required to translate these insights into practical applications. Here, we discuss how these classes of uncertainty serve as a first point of orientation for further exploration, visualization, and communication of the uncertainty analysis to support decision-making. Although our study primarily uses a variance-based approach to characterize output uncertainty, we acknowledge its limitations in capturing skewed distributions and suggest that future research could benefit from density-based approaches for a more nuanced understanding of risk (cf. Uncertainty and sensitivity analysis section in Materials and Methods) (*17*).

In our study, most input factors for uncertainty and sensitivity analysis represent forms of epistemic uncertainty. Within the context of climate-related assessments, we discuss between two primary types of epistemic uncertainty: scenario uncertainty and model uncertainty. Scenario uncertainty pertains to the unpredictability of future pathways, such as greenhouse gas emissions, stemming from various potential trajectories of human activities (*20*–*23*). Model uncertainty, conversely, reflects the limitations in our ability to represent complex systems, including discrepancies between models and the actual climate system they aim to simulate (*20*–*22*). While these two types of epistemic uncertainty differ fundamentally in their characteristics and origins, our framework allows for their evaluation within the same quantitative analysis.

Model uncertainty plays a substantial role in our analysis and can be examined at multiple levels. At the overall risk model level, the selection among different TC models (MIT, CHAZ, STORM, and IBTrACS_p) represents a source of model uncertainty, stemming from the varied approaches and assumptions inherent in each TC model. When considering individual risk model components, we observe varying degrees of model uncertainty. In the hazard component, model uncertainty is substantial, particularly for hazard-related input factors like the GCM choice (GCM) and TCGI formulation (TCGI moisture variable). For the exposure component, model uncertainty related to the GDP model choice (GDP model) is comparatively small (*20*–*22*). Scenario uncertainty is evident in varying hazard emission scenarios (SSP hazard), showing minor influence on the output uncertainty of TC risk change estimates across models. Conversely, scenario uncertainty of exposure, indicated by the SSP-based scaling factors for GDP growth (SSP exposure), is a key source of uncertainty in a wide range of outputs (Sensitivity of future TC risk change section).

The reducibility of these uncertainties varies. Model uncertainty, particularly in the hazard component, is theoretically reducible through model refinement, enhanced data collection, and focused research (*19*, *22*, *25*, *64*). In contrast, scenario uncertainty, which is inherently tied to future human choices, cannot be reduced in the same way. In the context of hazard modeling, scenario uncertainty may hold secondary importance due to its observed low sensitivity. However, exposure-related scenario uncertainty is high and thus becomes critically relevant from a decision-making standpoint. Although scenario uncertainty cannot be reduced, it can motivate decision-makers to favor scenarios of minimal risk. Specifically, the importance of scenario uncertainty in the exposure component (SSP exposure) may motivate decision-makers to choose policy options that accommodate anticipated population and economic growth in areas and timescales less exposed to TC hazards. For instance, Geiger *et al.* (*65*) found that limiting global warming to 2°C by 2100, as opposed to reaching the same temperature by 2050, can substantially reduce the population exposed to TC risks, demonstrating how scenario choices in socioeconomic and climate mitigation strategies critically affect future TC exposure. While Geiger *et al.* (*65*) focused on exposed populations, the same conclusions apply to exposed asset values, as shown in the present study or any other type of exposure. As an illustration, fig. S12 separates the uncertainty output of this study by scenarios and visually distinguishes between matched and mismatched combinations of socioeconomic and emission pathways, offering a clearer orientation for interpreting how scenario-specific choices influence risk outcomes. Therefore, policy decisions that strategically manage the exposure of people or assets to TCs and other hazards can effectively reduce future risks, thereby guiding decisions subject to scenario uncertainty.

Beven *et al.* (*66*) reviewed the treatment of epistemic uncertainty across various natural hazards and found that there is no consistent approach to dealing with epistemic uncertainty. They emphasized that epistemic uncertainties are often treated as aleatory ones, using specified distributional forms, which can lead to an underestimation of potential uncertainty in risk assessments and, consequently, a lack of robustness in decision-making and preparedness for future surprises. In the context of our study and climate risk assessment more broadly, recognizing and properly accounting for epistemic uncertainties is crucial. We thus represent different input factors that constitute forms of epistemic uncertainty by selecting from a list of scientifically justified inputs based on alternative representations of future climate and socioeconomic systems rather than defining a set of additive or multiplicative perturbation factors for each input factor whenever possible, mirroring previous studies by Dawkins *et al.* (*31*) and Meiler *et al.* (*30*). However, we also proactively communicate the potential for blind spots in epistemic uncertainty assessment. This acknowledgment and transparency are vital for understanding the limitations of current epistemic uncertainty assessments, better managing these inherent limitations, and improving the robustness and reliability of climate risk model output. This approach ensures that risk assessments remain relevant and actionable, even in the face of substantial uncertainty.

Aleatory uncertainty is represented in the present study in the event subsampling of the hazard sets. Through sensitivity analysis, we observe divergent responses to subsampling (Event subsampling base/future) across different hazard models (Fig. 4 and table S2). Specifically, the statistical-dynamical models MIT and CHAZ show no sensitivity to event subsampling, suggesting that they may inherently capture natural variability through their physics-based methodologies and the generation of new event sets for future climates. In contrast, the purely statistical models, IBTrACS_p and STORM, exhibited sensitivity to subsampling. This indicates that these models, which have the historical track sets at their foundation, may require the inclusion of a subsampling step to represent aleatory uncertainty adequately. Further validation is needed to strengthen this conclusion, however.

Despite its nonreducible nature (*67*), quantifying aleatory uncertainty is crucial, as demonstrated by the event subsampling in this study. Moreover, and perhaps counterintuitively, while aleatory uncertainty is nonreducible (e.g., it is not even in principle possible to forecast the weather in 20 years due to the chaotic nature of the Earth system), it can be accurately represented in the form of a probability distribution. This differentiates it from epistemic uncertainty, which often is inherently indeterminate or not readily quantifiable (*66*). Accurately quantifying aleatory uncertainty thus helps differentiate it from epistemic uncertainty, guiding research efforts more effectively toward understanding and modeling the complex behaviors of natural systems.

Normative uncertainty is often interrelated with the other categories but also extends beyond the focus of this paper into implications for how modeling results are used for societal decisions. Considering normative aspects in the context of scenario uncertainty in TC risk assessment, it is crucial to consider a wide range of scenarios to avoid blind spots in risk assessment. Unlike in policymaking, where particular scenarios or targets often represent a (normatively) favored developmental path (such as the Paris Agreement), excluding specific scenarios a priori in a risk setting could result in either under- or overestimation of risk. Furthermore, caution is also advised when considering whether to weight some scenarios as more likely than others, or to weight all scenarios equally, as improper weighting could exacerbate the risk of over- or underestimation.

Concerning normative facets in model uncertainty, the concept of “fitness for purpose” is vital (*21*). As a simple example, specifying risk only as EAD based on property values would tend to divert attention toward luxury coastal villas in Florida and away from informal coastal settlements in Bangladesh, although TC impacts on the latter would have a much more negative effect on people’s well-being. EAD would be an appropriate risk metric for estimating potential insurance payouts, whereas for humanitarian goals, risk metrics such as the number of people in poverty affected would be more appropriate for the modeling purpose. Hence, different stakeholders and sectors require varied outputs from TC hazard models and different calculations of risk. Given practical constraints, selecting specific models early in the risk assessment process is often necessary, but this choice substantially influences the results. Indeed, as highlighted in our findings (Sensitivity of future TC risk change section), looking at the relative change or absolute impacts of TCs completely changes the narrative, not only in terms of the output values themselves (e.g., a few percent versus several billion USD) but also in the sensitivity to input uncertainties (e.g., the dominance of hazard- or exposures. Impact function-related factors in Fig. 4 versus fig. S6). Hence, a bottom-up approach, incorporating stakeholder needs, goals and values to guide model selection, is recommended for tailoring risk assessments effectively (*16*).

Understanding the different types of uncertainties—aleatory, epistemic, and normative—is vital for risk modeling and informed decision-making. Linking these types of uncertainty to systematic uncertainty and sensitivity quantification across different TC hazard models, this study offers a nuanced view of TC risk assessment, which can guide future research and provide decision-critical insights. In particular, key findings here are that the range of uncertainty in TC risk change is strongly model dependent, and further that which components of the modeling chain introduce the greatest sources of uncertainty also varies depending on choices in other components of that chain. This indicates that not only is the uncertainty itself uncertain but so are which factors are most responsible for that uncertainty. Since our multimodel ensemble is small—with only four hazard models, for example—this raises the possibility that adding more models could change the conclusions quantitatively or perhaps even qualitatively. We suspect that this situation is not unique to TCs but may also apply to other aspects of climate risk. This suggests that humility in the use and interpretation of quantitative climate risk models is warranted, and that adaptation decisions should be based on multiple lines of evidence.

We also advocate for increased research on exposure and vulnerability modeling. While our uncertainty and sensitivity analysis might not explicitly highlight this need, we assert that this could be in part because these components are represented in a simple and reduced way in available datasets and the current modeling setup and fewer options are available to bracket the possibilities and define the uncertainties. The fact that exposure and vulnerability have been much less studied in forms that can readily be input into such modeling approaches (at least in the public domain) than the hazard offers immediate opportunities for impactful research, as these areas have a pronounced influence on results. While there have been some initial efforts to develop gridded population (*68*) and GDP projections (*69*), these efforts are still in their early stages. In this study, exposure projections are based on uniform SSP-derived GDP growth factors. These were not designed to be used in a spatially explicit fashion (*43*) and fail to capture the spatial nuances of socioeconomic development, such as urbanization patterns. We note that the risk values reported in this study may be dominated by high-GDP areas because the risk metrics are aggregated over the four, large-scale regions (see Study regions section in Materials and Methods). For local studies, higher resolution and the best available local data would be required.

Furthermore, our approach of decoupling socioeconomic pathways from their associated climate projections deviates from the original intent of the SSP framework, potentially leading to results that are challenging to interpret within individual SSP contexts. However, this decoupling is crucial for comprehensive risk assessment. It allows us to assess uncertainty within the SSP framework itself, considering scenarios where socioeconomic pathways might lead to unexpected climate outcomes (e.g., an SSP2 pathway resulting in an SSP3-7.0 climate). While this approach may not be directly applicable in policy contexts requiring scenario consistency, it provides valuable insights for risk management by capturing a broader spectrum of uncertainties and avoiding blind spots that could arise from rigid adherence to predefined pathway-climate combinations.

For vulnerability, no viable options exist for simulating changes in future vulnerabilities at scale. Hence, achieving projections of exposures and vulnerabilities projections of exposures and vulnerabilities in spatially explicit forms that match the complexity of climate hazards demands an effort comparable to that of global climate modeling, encompassing both social development and adaptation strategies.

Providing reliable TC risk assessment, including uncertainty and sensitivity analysis, is important for emerging fields like physical climate risk disclosure (*3*, *4*) or changing traditional sectors like insurance. In both cases, rules by which climate risk science can be used appropriately to inform climate risk assessment have not yet been developed or are changing. As we move forward, it is essential to refine our models continually, choose models according to their application, and be critically aware of the normative assumptions that underlie our assessments. The type of uncertainty quantification and attribution we performed in our study is based on open models, which we believe is an important prerequisite for transparent and reliable TC risk assessment. The academic realm has seen a shift toward more open models and data, a trend that is much needed and that we hope the industry will follow. Ultimately, we aim to balance risk assessments that are both accurate and actionable.

## MATERIALS AND METHODS

In this study, we use the open-source, probabilistic climate risk modeling framework CLIMADA (*15*) to assess future TC risk changes and quantify and attribute related uncertainties across four main regions. Several key components underpin this methodology. Specifically, TC models (section TC models) generate synthetic TC track or event sets (section TC track sets). These track sets are converted to two-dimensional wind fields by parametric wind models, representing the TC hazard sets (section TC hazard). These hazard sets, along with asset exposure layers (section Asset exposure representations) and impact functions (section Impact functions), serve as inputs into CLIMADA (section Risk model CLIMADA). CLIMADA is then used to estimate TC risks and perform uncertainty and sensitivity analyses (section Uncertainty and sensitivity analysis). We describe these key components in more detail in the subsequent sections.

### Study regions

**Fig. 5.**
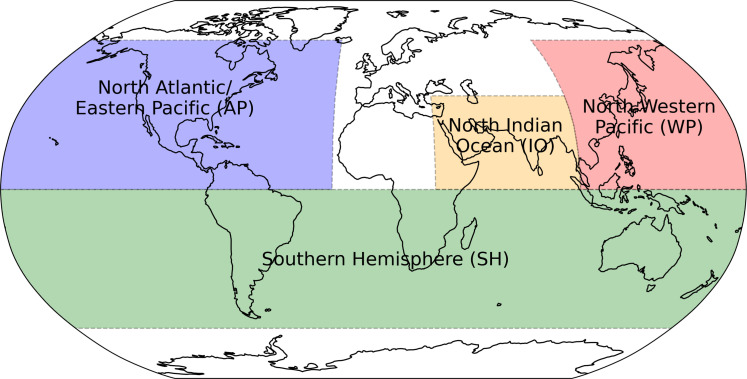
Global study regions. AP (blue), IO (orange), SH (green), and WP (red).

In this study, we assess future TC risk increases across four main regions, as shown in Fig. 5 and established in Meiler *et al.* (*16*). These regions are chosen to broadly reflect distinct TC areas, focusing on the impact on land. Hence, we combine TCs originating in the North Atlantic and Eastern Pacific (AP) into one region to evaluate the socioeconomic impact on national GDPs, accounting for countries with coastlines in multiple basins, such as the United States, Mexico, and Central American nations. Similarly, the Southern Hemisphere (SH) is treated as a unified region, with the North Indian Ocean (IO) and Western Pacific (WP) completing the geographical split.

### Risk model CLIMADA

The open-source, probabilistic climate risk model CLIMADA integrates climate and weather-related hazards with the exposure and vulnerability of assets, populations, and infrastructure on a global scale (*15*). Developed as a community initiative, its Python 3 source code is freely accessible under the GNU General Public License Version 3. In this study, we use the Python (3.9+) version of CLIMADA release v4.1.1 (*70*) to evaluate the projected increase in direct economic losses from TCs in the mid- and late-21st century, relative to a contemporary baseline. Damage estimates are calculated at a spatial resolution of 300 arc sec (approximately 10 km at the equator).

### TC hazard

In CLIMADA, the TC hazard is represented by a two-dimensional wind field created by integrating TC track sets with a parametric wind model. This study uses two distinct wind models based on the parameterizations from Holland (*71*) and Emanuel and Rotunno (*72*), applied to all TC track sets described in TC track sets section. These wind models calculate the gridded 1-min average sustained winds at 10 m above ground, comprising both a circular wind field component and the translational wind speed generated by the TC’s movement. A key difference between the models lies in how they compute the (absolute) angular velocity from the wind profile. In both models, an attenuation factor, as suggested by Geiger *et al.* (*9*), is used to model the reduction of the translational wind component with distance from the cyclone center. For this study, wind fields are computed at a resolution of 300 arc sec. CLIMADA uses the peak lifetime wind speed at each location as the hazard variable, disregarding values below 34 knots (17.5 m/s).

### TC models

Different synthetic TC models exist, each with their unique modeling approach that influences the resulting TC event sets. Prominent methods commonly used for TC risk assessment are either purely statistical (*39*, *40*) or coupled statistical-dynamical (*35*–*38*). Here, we briefly review the key similarities and differences of the global, academically available TC models used in this study.

Statistical-dynamical TC models like the MIT (*35*, *36*) and CHAZ (*37*, *38*) both use dynamical downscaling of TC tracks from reanalyses or climate model output. These models follow the three-step process of genesis, track, and intensity modeling. The main genesis mechanism of the MIT model is random seeding and natural selection (*35*, *36*), while CHAZ uses a TCGI (*37*, *38*), which statistically links the occurrence of TCs to large-scale environmental conditions favorable for TC development. TC tracks are propagated via synthetic local winds from a beta-and-advection model (*73*) in both models. Intensity changes along the tracks are simulated using a dynamical model (MIT) (*35*, *36*) or an autoregressive model using physics-based drivers (CHAZ) (*37*, *38*).

In contrast, the fully statistical, global, open-source model STORM (*39*, *40*) uses autoregressive formulas to simulate both the track and intensity of a TC. STORM run for present-day TC activity uses data from IBTrACS (*42*) and ECMWF’s ERA-5 reanalysis (*74*) for input, generating synthetic TCs with characteristics consistent with observed statistics. For future climate simulations, Bloemendaal *et al.* (*40*) derived changes in key TC variables from four high-resolution GCM simulations (1979–2014 versus 2015–2050) and applied these to TC variables from historical data. On this basis, they ran STORM to simulate future TC activity under climate change.

The fourth TC modeling approach featured in this study is the generation of probabilistic TC tracks from the IBTrACS records (*42*). This approach, embedded in the CLIMADA platform (*15*) uses a simple interpolation method based on a random-walk process (*12*, *41*). The method generates a probabilistic track distribution from historical observations without explicitly incorporating detailed physical, climate, or basin-specific characteristics. A more detailed description can be found in the Supplementary Materials of Gettelman *et al.* (*12*), and the handling of observations from the IBTrACS record (*42*) is detailed in Meiler *et al.* (*16*). Similar to STORM, the probabilistic IBTrACS obtained from the CLIMADA platform can be climate conditioned by changing their frequency and intensity according to scaling factors derived by Knutson *et al.* (*75*) for the CMIP5 generation of climate models. This approach is simpler than the future climate STORM modeling approach (*40*). Instead of rerunning a TC model based on several scaled key TC variables, it just applies scaling factors to hazard intensity and frequency. We note that, to date, climate-conditioned IBTrACS are not available for the newest generation of climate models (CMIP6). Furthermore, the resulting future TC event sets from the STORM model and probabilistic, climate-conditioned IBTrACS do not contain spatial variations compared to their present-day counterparts. In comparison, future MIT and CHAZ hazard sets are completely new event sets, including spatial variations of the tracks.

### TC track sets

In this study, the MIT TC model (*35*, *36*) was used to generate TC track sets from input of nine distinct GCMs (detailed in table S4) under three emission scenarios: SSP245, SSP370, and SSP585, which are part of the CMIP6 generation. The model simulations cover three time frames: the present-day reference period (1995–2014), a mid-century period (2041–2060), and a late-century period (2081–2100). The model generated 500 TCs each year within these periods using the three-step process of genesis, track, and intensity modeling described in the previous section. The annual variation in the number of TCs is influenced by the specific boundary conditions set by the GCMs, such as potential intensity and wind shear, affecting how many of the initial seeds develop into full TCs. The model uses a three-step process of genesis, track, and intensity modeling to generate 500 TCs each year. The development of initial seeds into full TCs is influenced by specific boundary conditions set by the GCMs, such as potential intensity and wind shear. To determine the final yearly TC frequency, a bias correction process is applied. This process compares the number of seeds that develop into full TCs under the specific boundary conditions to the calibrated total of 500 events per year. The ratio of developed TCs to total seeds is then used to adjust the final TC count, ensuring that it reflects the expected frequency given the climate conditions represented in each simulation.

CHAZ (*37*, *38*) was used to generate TC event sets for three emission scenarios (SSP245, SSP370, and SSP585) drawing from six (CESM2, CNRM-CM6-1, EC-Earth3, IPSL-CM6A-LR, MIROC6, and UKESM1-0-LL) of the nine CMIP6 GCMs also used by the MIT model (cf. table S4) and two distinctly different choices of moisture variable used in the TCGI component of CHAZ (*38*). CHAZ is downscaled for every combination of emission scenario, GCM, and TCGI with 10 different realizations of the genesis model and resulting tracks. For each genesis realization, 40 ensembles of the intensity model are produced. In this study, we use all 10 genesis ensembles but randomly select 8 of the 40 intensity ensembles. This results in 80 ensemble members, reducing computational costs while maintaining a sufficiently large track set for robust TC risk assessments (*16*).

Analogous to the MIT hazard sets, we contrast TC event sets for a present climate reference state (1995–2014) with two future periods: mid-century (2041–2060) and end of the century (2081–2100). In addition, CHAZ hazard sets require a frequency bias correction (*16*, *37*, *76*). We adjust the hazard frequency of all reference state hazard sets using the observed frequencies in each basin. This adjustment is done by comparing the model-generated TC frequencies with observed frequencies and applying a correction factor based on the difference. Numbers for the observed IBTrACS genesis events are derived from Bloemendaal *et al.* (table 3) (*39*) and are combined to values relevant to the study regions of this manuscript (Fig. 5). Each TC in the baseline hazard set is adjusted to ensure that the overall frequency aligns with the observed average. This adjusted frequency is then applied to the TCs in the future climate hazard sets. In our approach, each future TC is assigned the same probability of occurrence as its present-day counterpart. However, the overall frequency of the event set (expressed as the number of TCs per year) may change because of variations in the total storm count, thereby reflecting potential changes in TC frequency under future climate conditions.

TC track sets from the statistical model STORM were used as released by Bloemendaal *et al.* (*39*, *40*) representing 10,000 years of present-day (1980–2018) (*39*) and future (SSP585; 2015–2050) synthetic TCs from an ensemble of four high-resolution climate models. Note that future STORM TC tracks are only available for a single emission scenario (SSP585) and the middle-of-the-century time period.

Finally, using the random walk algorithm of the CLIMADA platform as described in the previous section, we generated a set of 24 probabilistic tracks for each observed TC between 1990 and 2010 for this study. Upon generating wind fields from these tracks (cf. TC hazard section) using two different parametric wind models (*71*, *72*), the hazard sets are climate-conditioned by applying constant, basin-specific factors to the tracks’ intensity and frequency. These factors were derived from the meta-analysis by Knutson *et al.* (*75*) summarizing the effects of climate change on TCs by CMIP5 climate models under RCP4.5 projections for the late-21st century. A linear scaling approach is used to estimate parameters for different future periods and the other three RCP scenarios (2.6, 6.0, and 8.5) according to the RCP database (*77*). Note that we did not generate climate-conditioned hazard sets for the RCP8.5 scenario at the end of the century as the current implementation of the respective module on the CLIMADA platform produces erroneous negative frequencies. In the remainder of this study, we refer to hazard sets generated via this approach as IBTrACS_p.

### Asset exposure representations

We generated a spatially explicit, gridded dataset of asset exposure values using the LitPop method. This approach disaggregates national asset value totals to grid cells based on a combination of nightlight intensity (Lit) and population density (Pop), as proposed by Eberenz *et al.* (*78*). The reference exposure layer for the present day is computed at a resolution of 300 arc sec using the GDP values (in USD) from 2005, approximately centered on the present-day TC track set periods. For future exposure representations—identical to Meiler *et al.* (*30*, *32*)—we use economic growth factors from the SSPs to approximate socioeconomic development, drawing from the SSP database that documents quantitative projections of SSPs and related scenarios (*43*). SSPs outline five potential trajectories for global changes in population, economic growth, technology, governance, and social norms over the next century, with a focus here on GDP projections as a measure of economic development. Three alternative GDP interpretations by the OECD (*44*), the IIASA (*45*), and the PIK (*46*) are considered, which, despite being based on the same SSP assumptions for economic growth determinants, vary in methods and results. We specifically query GDP growth factors for 2050 and 2090 for each country across all five SSPs from these models, scaling the reference asset values accordingly for the two future time periods across all scenarios. In this approach, the spatial distribution of assets remains static, not accounting for potential spatial shifts in socioeconomic factors.

### Impact functions

In risk assessment, impact functions represent vulnerability, describing how hazard intensity translates to damage on assets. In this study, we use regionally calibrated impact functions as developed by Eberenz *et al.* (*47*). These functions are fitted to nine different global regions, reflecting the diverse vulnerability levels across the world. For this study, we applied the same impact functions to all four synthetic TC track sets. In contrast to the well-developed methodologies for exposure and particularly hazard modeling, no viable options exist for simulating changes in future vulnerabilities. Therefore, we do not hypothesize about changes to the vulnerability function in the future but test uncertainties by varying the vulnerability function’s slope parameter of regionally calibrated vulnerability functions (*47*) across a wide range. This ensures that the calibration reflects the specific socioeconomic and vulnerability characteristics of each region.

### Uncertainty and sensitivity analysis

For this study’s uncertainty and sensitivity quantification, we use the unsequa module on the CLIMADA platform (*13*). We extended the unsequa module to compute uncertainties for changes in risk directly. These functionalities are now also publicly available as “CalcDeltaImpact” in CLIMADA v.4.1.1 or higher. The module seamlessly integrates the *SALib* Python package (*79*) and allows for uncertainty and sensitivity analyses of all CLIMADA-based risk calculations. A central aspect of uncertainty and sensitivity analysis is determining input factors and characterizing their variability space (*13*, *17*, *29*). This section delineates our approach to address uncertainties in inputs related to (future) TC hazards, exposure, and vulnerability within the context of our study (Fig. 1).

We choose from a discrete list of scientifically justified alternative versions of future climate and socioeconomic systems. We prioritize this approach over defining additive or multiplicative perturbations for each input factor because it avoids the challenges of defining perturbations without relevant information, directly relates the output to chosen input combinations, and circumvents assumptions about the likelihood of specific input scenarios. Specifically, we define five input factors characterizing the hazard components, three for the exposure and one for the impact function (see Table 1). For event subsampling, targeting the aleatory uncertainty of the hazard set, we favor continuous sampling to better represent its inherent variability. Continuous sampling is also used for the parameters describing the impact function due to the absence of a scientifically supported discrete alternative.

**Table 1.** Input factors and their variability space. The first column lists all input factors of the uncertainty and sensitivity analysis, indicating which risk model component they relate to. Variable names, as referred to in the text and figures of this study, are listed in the second column. The type of the parameter range is indicated in the third column. The actual parameter ranges for each hazard model are detailed in the last four columns.

Input factor	Variable name	Type	Range			
			MIT	STORM	CHAZ	IBTrACS_p*
Hazard: GCM	GCM	Discrete	CESM2, CNRM6, EC-Earth, FGOALS, IPSL6, MIROC6, MPI2, MRI6, UKESM**	CMCC, CNRM, EC-Earth, HadGEM3 (*40*)	CESM2, CNRM6, EC-Earth, IPSL6, MIROC6, UKESM**	N/A
Hazard: emission scenario	SSP hazard	Discrete	SSP2-4.5, SSP3-7.0, SSP5-8.5	N/A (STORM is only available for SSP5-8.5, thus not sampled)	SSP2-4.5, SSP3-7.0, SSP5-8.5	RCP2.6, RCP4.5, RCP6.0, RCP8.5 (RCP8.5 not available for 2100)
Hazard: wind model	Wind model	Discrete	Holland (*70*), Emanuel and Rotunno (*71*)	Holland (*70*), Emanuel and Rotunno (*71*)	Holland (*70*), Emanuel and Rotunno (*71*)	Holland (*70*), Emanuel and Rotunno (*71*)
Hazard: moisture variable TCGI	Moisture variable TCGI	Discrete	N/A	N/A	CRH, SD (*38*)	N/A
Hazard: bootstrapping	Event subsampling base/future	Continuous	80% of every year	1 of 10 ensembles	80% of event set	80% of event set
Exposure: SSP-based GDP scaling	SSP exposure	Discrete	SSP1, SSP2, SSP3, SSP4, SSP5			
Exposure: GDP model	GDP model	Discrete	OECD (*44*), IIASA (*45*), PIK (*46*)			
Exposure: m,n scaling LitPop	Exposure urban/rural weighting	Discrete	*m* = (0.5, 1.0, 1.5), *n* = (0.5, 1.0, 1.5)			
Impact functions	Vulnerability function midpoint	Continuous	Within IQR of regional TC calibration (*47*)			

*CMIP5

**Full GCM names and references are in table S4

We generate a set of *N* = 2^10^ (1024) samples using the Sobol sampling algorithm (*53*, *54*). Each sample represents a unique set of values for the input factors listed in Table 1 and shown in Fig. 1. The Sobol method requires a specific number of input factor combinations, calculated as *N* ·(*k* + 2), where *k* is the number of input factors. The total number of input factor combinations varies from 18,432 to 22,528, depending on whether 8, 9, or 10 input factors are considered in each specific analysis. This variation is due to differences in factor availability across the TC models. On average, this results in approximately 20,000 input factor combinations per analysis. This number of combinations provides sufficient coverage for the uncertainty analysis to converge, meaning that additional samples would not notably change the results.

For each sample, we perform a risk calculation. This process yields a distribution of risk values rather than a single deterministic result. In our study, we focus on two risk metrics: the change in EAD and the change in the 100-year event damage. By repeating this calculation across all samples, we generate distributions for both of these risk metrics. These output distributions underpin the uncertainty analysis and initiate the sensitivity analysis. Using the Sobol quasi-Monte Carlo sequence (*53*), we present first- and total-order sensitivity indices to estimate each input factor’s contribution to output variance. Specifically, the first-order sensitivity index measures the direct impact of a single input factor on the output uncertainty, independent of other factors. The total-order sensitivity index, on the other hand, captures both the direct effects and any potential interactions with other input parameters. Together, these indices provide a comprehensive view of how changes in input variables influence the uncertainty in our results.

While our study primarily uses a variance-based approach to characterize the uncertainty in our output distributions, we recognize that variance alone may not fully capture the nature of skewed distributions, which are common in hazard contexts. Variance provides a measure of spread but does not convey information about the asymmetry or tails of the distribution, which are crucial in understanding the full extent of risk. Although this approach is suitable for addressing the specific questions in this study, future research could benefit from exploring density-based approaches (*17*). These methods examine the entire probability density function of the output, providing a more nuanced view of output uncertainty by capturing skewness and the presence of long tails. Incorporating density-based approaches could enhance the robustness of uncertainty characterization, offering deeper insights into the nature of the distributions and potentially leading to more informed decision-making in hazard risk assessments.

## Supplementary Materials

### The PDF file includes:

Supplementary Discussion Figs. S1 to S12 Tables S1 to S5 Legend for table S6 References

## References

[1] J. M. Keenan, A climate intelligence arms race in financial markets. Science 365, 1240–1243 (2019). 31604223 10.1126/science.aay8442

[2] M Condon, Climate services: The business of physical risk (2023).

[3] A. Arribas, R. Fairgrieve, T. Dhu, J. Bell, R. Cornforth, G. Gooley, C. J. Hilson, A. Luers, T. G. Shepherd, R. Street, N. Wood, Climate risk assessment needs urgent improvement. Nat. Commun. 13, 4326 (2022). 36008398 10.1038/s41467-022-31979-w PMC9411596

[4] T. Fiedler, A. J. Pitman, K. Mackenzie, N. Wood, C. Jakob, S. E. Perkins-Kirkpatrick, Business risk and the emergence of climate analytics. Nat. Clim. Change 11, 87–94 (2021).

[5] L. M. Braman, P. Suarez, M. K. van Aalst, Climate change adaptation: Integrating climate science into humanitarian work. Intern. Rev. Red Cross 92, 693–712 (2010).

[6] L. Jones, A. Dougill, R. G. Jones, A. Steynor, P. Watkiss, C. Kane, B. Koelle, W. Moufouma-Okia, J. Padgham, N. Ranger, J.-P. Roux, P. Suarez, T. Tanner, K. Vincent, Ensuring climate information guides long-term development. Nat. Clim. Change 5, 812–814 (2015).

[7] E. C. de Perez, S. J. Mason, Climate information for humanitarian agencies: Some basic principles. Earth Perspect. 1, 11 (2014).

[8] M. Enenkel, A. Kruczkiewicz, The humanitarian sector needs clear job profiles for climate science translators now more than ever. Bull. Am. Meteorol. Soc. 103, E1088–E1097 (2022).

[9] T. Geiger, K. Frieler, D. N. Bresch, A global historical data set of tropical cyclone exposure (TCE-DAT). Earth Syst. Sci. Data 10, 185–194 (2018).

[10] M. Berlemann, D. Wenzel, Hurricanes, economic growth and transmission channels: Empirical evidence for countries on differing levels of development. World Dev. 105, 231–247 (2018).

[11] R. Mendelsohn, K. Emanuel, S. Chonabayashi, L. Bakkensen, The impact of climate change on global tropical cyclone damage. Nat. Clim. Change 2, 205–209 (2012).

[12] A. Gettelman, D. N. Bresch, C. C. Chen, J. E. Truesdale, J. T. Bacmeister, Projections of future tropical cyclone damage with a high-resolution global climate model. Clim. Change 146, 575–585 (2018).

[13] C. M. Kropf, A. Ciullo, L. Otth, S. Meiler, A. Rana, E. Schmid, J. W. McCaughey, D. N. Bresch, Uncertainty and sensitivity analysis for probabilistic weather and climate-risk modelling: An implementation in CLIMADA v.3.1.0. Geosci. Model Dev. 15, 7177–7201 (2022).

[14] C.B. Field, V. Barros, T.F. Stocker, D. Qin, D.J. Dokken, K.L. Ebi, M.D. Mastrandrea, K. J. Mach, G.-K. Platner, S. K. Allen, M. Tignor, P. M. Midgley, IPCC, Managing the Risks of Extreme Events and Disasters to Advance Climate Change Adaptation. A Special Report of Working Groups I and II of the Intergovernmental Panel on Climate Change. (2012).

[15] G. Aznar-Siguan, D. N. Bresch, CLIMADA v1: A global weather and climate risk assessment platform. Geosci. Model Dev. 12, 3085–3097 (2019).

[16] S. Meiler, T. Vogt, N. Bloemendaal, A. Ciullo, C.-Y. Lee, S. J. Camargo, K. Emanuel, D. N. Bresch, Intercomparison of regional loss estimates from global synthetic tropical cyclone models. Nat. Commun. 13, 6156 (2022). 36257997 10.1038/s41467-022-33918-1 PMC9579140

[17] F. Pianosi, K. Beven, J. Freer, J. W. Hall, J. Rougier, D. B. Stephenson, T. Wagener, Sensitivity analysis of environmental models: A systematic review with practical workflow. Environ. Model. Software 79, 214–232 (2016).

[18] T. Wagener, R. Reinecke, F. Pianosi, On the evaluation of climate change impact models. WIREs Clim. Change 13, e772 (2022).

[19] W. Walker, P. Harremoës, J. Rotmans, J. van der Sluijs, M. van Asselt, P. Janssen, M. Krayer von Krauss, Defining uncertainty: A conceptual basis for uncertainty management in model-based decision support. Integ. Assessment 4, 5–17 (2003).

[20] E. Hawkins, R. Sutton, The potential to narrow uncertainty in regional climate predictions. Bull. Am. Meteorol. Soc. 90, 1095–1108 (2009).

[21] W. S. Parker, Predicting weather and climate: Uncertainty, ensembles and probability. Stud. Hist. Philos. Sci. B Stud. Hist. Philos. Mod. Phys. 41, 263–272 (2010).

[22] R. Knutti, Climate model confirmation: From philosophy to predicting climate in the real world, in *Climate Modelling: Philosophical and Conceptual Issues*, E. A. Lloyd and E. Winsberg, Eds. (Springer International Publishing, 2018), pp. 325–359.

[23] R. H. Moss, J. A. Edmonds, K. A. Hibbard, M. R. Manning, S. K. Rose, D. P. van Vuuren, T. R. Carter, S. Emori, M. Kainuma, T. Kram, G. A. Meehl, J. F. B. Mitchell, N. Nakicenovic, K. Riahi, S. J. Smith, R. J. Stouffer, A. M. Thomson, J. P. Weyant, T. J. Wilbanks, The next generation of scenarios for climate change research and assessment. Nature 463, 747–756 (2010). 20148028 10.1038/nature08823

[24] R. Bradley, M. Drechsler, Types of uncertainty. Erkenntnis 79, 1225–1248 (2014).

[25] R. Bradley, K. Steele, Making climate decisions. Philos. Compass 10, 799–810 (2015).

[26] L. A. Mayer, K. Loa, B. Cwik, N. Tuana, K. Keller, C. Gonnerman, A. Parker, R. J. Lempert, Understanding scientists’ computational modeling decisions about climate risk management strategies using values-informed mental models. Glob. Environ. Change 42, 107–116 (2017).

[27] S. O. Hansson, Evaluating the uncertainties. *The Argumentative Turn in Policy Analysis: Reasoning about Uncertainty*, S. O. Hansson, G. Hirsch Hadorn, Eds. (Logic, Argumentation & Reasoning, Springer International Publishing, 2016), pp. 79–104.

[28] A. Saltelli, M. Ratto, T. Andres, F. Campolongo, J. Cariboni, D. Gatelli, M. Saisana, S. Tarantola, *Global Sensitivity Analysis: The Primer* (John Wiley & Sons Ltd, 2008).

[29] A. Saltelli, K. Aleksankina, W. Becker, P. Fennell, F. Ferretti, N. Holst, S. Li, Q. Wu, Why so many published sensitivity analyses are false: A systematic review of sensitivity analysis practices. Environ. Model. Softw. 114, 29–39 (2019).

[30] S. Meiler, A. Ciullo, C. M. Kropf, K. Emanuel, D. N. Bresch, Uncertainties and sensitivities in the quantification of future tropical cyclone risk. Commun. Earth Environ. 4, 371 (2023).

[31] L. C. Dawkins, D. J. Bernie, F. Pianosi, J. A. Lowe, T. Economou, Quantifying uncertainty and sensitivity in climate risk assessments: Varying hazard, exposure and vulnerability modelling choices. Clim. Risk Manag. 40, 100511 (2023).

[32] S. Meiler, A. Ciullo, D. N. Bresch, C. M. Kropf, Uncertainty and sensitivity analysis for probabilistic, global modelling of future tropical cyclone risk, *14th International Conference on Applications of Statistics and Probability in Civil Engineering (ICASP14)* (2023). 10.25546/103244.

[33] S. Lo Piano, R. Sheikholeslami, A. Puy, A. Saltelli, Unpacking the modelling process via sensitivity auditing. Futures 144, 103041 (2022).

[34] T. Page, P. Smith, K. Beven, F. Pianosi, F. Sarrazin, S. Almeida, L. Holcombe, J. Freer, N. Chappell, T. Wagener, Technical note: The CREDIBLE Uncertainty Estimation (CURE) toolbox: Facilitating the communication of epistemic uncertainty. Hydrol. Earth Syst. Sci. 27, 2523–2534 (2023).

[35] K. Emanuel, S. Ravela, E. Vivant, C. Risi, A statistical deterministic approach to hurricane risk assessment. Bull. Am. Meteorol. Soc. 87, 299–314 (2006).

[36] K. Emanuel, The hurricane—Climate connection. Bull. Am. Meteorol. Soc. 89, ES10–ES20 (2008).

[37] C.-Y. Lee, M. K. Tippett, A. H. Sobel, S. J. Camargo, An environmentally forced tropical cyclone hazard model. J. Adv. Model. Earth Syst. 10, 223–241 (2018).

[38] C.-Y. Lee, S. J. Camargo, A. H. Sobel, M. K. Tippett, Statistical–dynamical downscaling projections of tropical cyclone activity in a warming climate: Two diverging genesis scenarios. J. Clim. 33, 4815–4834 (2020).

[39] N. Bloemendaal, I. D. Haigh, H. de Moel, S. Muis, R. J. Haarsma, J. C. J. H. Aerts, Generation of a global synthetic tropical cyclone hazard dataset using STORM. Sci. Data 7, 40 (2020). 32029746 10.1038/s41597-020-0381-2 PMC7005259

[40] N. Bloemendaal, H. de Moel, A. B. Martinez, S. Muis, I. D. Haigh, K. van der Wiel, R. J. Haarsma, P. J. Ward, M. J. Roberts, J. C. M. Dullaart, J. C. J. H. Aerts, A globally consistent local-scale assessment of future tropical cyclone risk. Sci. Adv. 8, eabm8438 (2022). 35476436 10.1126/sciadv.abm8438 PMC9045717

[41] S. Kleppek, V. Muccione, C. C. Raible, D. N. Bresch, P. Koellner-Heck, T. F. Stocker, Tropical cyclones in ERA-40: A detection and tracking method. Geophys. Res. Lett. 35, L10705 (2008).

[42] K. R. Knapp, M. C. Kruk, D. H. Levinson, H. J. Diamond, C. J. Neumann, The international best track archive for climate stewardship (IBTrACS). Bull. Am. Meteorol. Soc. 91, 363–376 (2010).

[43] K. Riahi, D. P. van Vuuren, E. Kriegler, J. Edmonds, B. C. O’Neill, S. Fujimori, N. Bauer, K. Calvin, R. Dellink, O. Fricko, W. Lutz, A. Popp, J. C. Cuaresma, S. Kc, M. Leimbach, L. Jiang, T. Kram, S. Rao, J. Emmerling, K. Ebi, T. Hasegawa, P. Havlik, F. Humpenöder, L. A. Da Silva, S. Smith, E. Stehfest, V. Bosetti, J. Eom, D. Gernaat, T. Masui, J. Rogelj, J. Strefler, L. Drouet, V. Krey, G. Luderer, M. Harmsen, K. Takahashi, L. Baumstark, J. C. Doelman, M. Kainuma, Z. Klimont, G. Marangoni, H. Lotze-Campen, M. Obersteiner, A. Tabeau, M. Tavoni, The shared socioeconomic pathways and their energy, land use, and greenhouse gas emissions implications: An overview. Glob. Environ. Change 42, 153–168 (2017).

[44] R. Dellink, J. Chateau, E. Lanzi, B. Magné, Long-term economic growth projections in the shared socioeconomic pathways. Glob. Environ. Change 42, 200–214 (2017).

[45] J. Crespo Cuaresma, Income projections for climate change research: A framework based on human capital dynamics. Glob. Environ. Change 42, 226–236 (2017).

[46] M. Leimbach, E. Kriegler, N. Roming, J. Schwanitz, Future growth patterns of world regions – A GDP scenario approach. Glob. Environ. Change 42, 215–225 (2017).

[47] S. Eberenz, S. Lüthi, D. N. Bresch, Regional tropical cyclone impact functions for globally consistent risk assessments. Nat. Hazard s Earth Syst. Sci. 21, 393–415 (2021).

[48] K. A. Emanuel, Global warming effects on U.S. hurricane damage. Weather Clim. Soc. 3, 261–268 (2011).

[49] K. M. Wilson, J. W. Baldwin, R. M. Young, Estimating tropical cyclone vulnerability: A review of different open-source approaches, in *Hurricane Risk in a Changing Climate*, J. M. Collins, J. M. Done, Eds. (Hurricane Risk, Springer International Publishing, 2022),pp. 255–281.

[50] C. Lemieux, *Monte Carlo and Quasi-Monte Carlo Sampling* (Springer Science & Business Media, 2009).

[51] C. Unterberger, P. Hudson, W. J. Botzen, K. Schroeer, K. W. Steininger, Future public sector flood risk and risk sharing arrangements: An assessment for Austria. Ecol. Econ. 156, 153–163 (2019).

[52] T. Knutson, S. J. Camargo, J. C. Chan, K. Emanuel, C. H. Ho, J. Kossin, M. Mohapatra, M. Satoh, M. Sugi, K. Walsh, L. Wu, Tropical cyclones and climate change assessment part II: Projected response to anthropogenic warming. Bull. Am. Meteorol. Soc. 101, E303–E322 (2020).

[53] I. M. Sobol, Global sensitivity indices for nonlinear mathematical models and their Monte Carlo estimates. Math. Comput. Simul. 55, 271–280 (2001).

[54] A. Saltelli, P. Annoni, I. Azzini, F. Campolongo, M. Ratto, S. Tarantola, Variance based sensitivity analysis of model output. Design and estimator for the total sensitivity index. Comput. Phys. Commun. 181, 259–270 (2010).

[55] K. Emanuel, Environmental factors affecting tropical cyclone power dissipation. J. Climate 20, 5497–5509 (2007).

[56] K. A. Emanuel, D. S. Nolan, Tropical cyclone activity and global climate, *26th Conference on Hurricanes and Tropical Meteorology* (Amer. Meteor. Soc., 2004), pp. 240–241.

[57] K. Emanuel, Tropical cyclone activity downscaled from NOAA-CIRES reanalysis, 1908–1958. J. Adv. Model. Earth Syst. 2, 1 (2010).

[58] E. D. Rappin, D. S. Nolan, K. A. Emanuel, Thermodynamic control of tropical cyclogenesis in environments of radiative-convective equilibrium with shear: Tropical cyclogenesis in variable climates. Q. J. Roy. Meteorol. Soc. 136, 1954–1971 (2010).

[59] A. H. Sobel, A. A. Wing, S. J. Camargo, C. M. Patricola, G. A. Vecchi, C.-Y. Lee, M. K. Tippett, Tropical cyclone frequency. Earth’s Future 9, e2021EF002275 (2021).

[60] A. Puy, P. Beneventano, S. A. Levin, S. Lo Piano, T. Portaluri, A. Saltelli, Models with higher effective dimensions tend to produce more uncertain estimates. Sci. Adv. 8, eabn9450 (2022). 36260678 10.1126/sciadv.abn9450 PMC9581491

[61] J. Roussos, R. Bradley, R. Frigg, Making confident decisions with model ensembles. Philos. Sci. 88, 439–460 (2021).

[62] M. Golnaraghi, P. Nunn, R. Muir-Wood, J. Guin, D. Whitaker, J. Slingo, G. Asrar, I. Branagan, G. Lemcke, C. Souch, M. Jean, A. Allman, M. Jahn, D. Bresch, P. Khalil, M. Beck, Managing Physical Risks of Climate: Leveraging Innovations in Catastrophe Risk Modelling: Reseach Brief, (November), 2018 (2018); https://api.semanticscholar.org/CorpusID:134139721.

[63] A. Ciullo, E. Strobl, S. Meiler, O. Martius, D. Bresch, Increasing countries’ financial resilience through global catastrophe risk pooling. Nat. Commun. 14, 922 (2023). 36808160 10.1038/s41467-023-36539-4 PMC9938104

[64] J. A. Curry, P. J. Webster, Climate science and the uncertainty monster. Bull. Am. Meteorol. Soc. 92, 1667–1682 (2011).

[65] T. Geiger, J. Gütschow, D. N. Bresch, K. Emanuel, K. Frieler, Double benefit of limiting global warming for tropical cyclone exposure. Nat. Clim. Change 11, 861–866 (2021).

[66] K. J. Beven, S. Almeida, W. P. Aspinall, P. D. Bates, S. Blazkova, E. Borgomeo, J. Freer, K. Goda, J. W. Hall, J. C. Phillips, M. Simpson, P. J. Smith, D. B. Stephenson, T. Wagener, M. Watson, K. L. Wilkins, Epistemic uncertainties and natural hazard risk assessment – Part 1: A review of different natural hazard areas. Nat. Hazards Earth Syst. Sci. 18, 2741–2768 (2018).

[67] M. Henrion, M. G. Morgan, The nature and sources of uncertainty, in *Uncertainty: A Guide to Dealing with Uncertainty in Quantitative Risk and Policy Analysis* (Cambridge Univ. Press, 1990), pp. 47–72.

[68] J. Gao, Global 1-km Downscaled Population Base Year and Projection Grids Based on the Shared Socioeconomic Pathways, Revision 01. NASA Socioeconomic Data and Applications Center (SEDAC), Palisades, New York (2020); 10.7927/q7z9-9r69; https://www.earthdata.nasa.gov/centers/sedac-daac.

[69] T. Wang, F. Sun, Global gridded GDP data set consistent with the shared socioeconomic pathways. Sci. Data 9, 221 (2022). 35589734 10.1038/s41597-022-01300-x PMC9120090

[70] G. Aznar-Siguan, E. Schmid, T. Vogt, S. Eberenz, C. B. Steinmann, T. Röösli, Y. Yu,E. Mühlhofer, S. Lüthi, I. J. Sauer, J. Hartman, C. M. Kropf, B. P. Guillod, Z. Stalhandske,A. Ciullo, D. N. Bresch, L. Riedel, C. Fairless, T. Schmid, P. M. M. Kam, N. Colombi, wjan262, S. Meiler, leonie-villiger, climada-jenkins, Rachel_B, raphael-portmann, veronicabozzini, DarioStocker, and scem, CLIMADA-project/climada_python: v4.0.1. Zenodo (2023); 10.5281/zenodo.8383171, doi:10.5281/zenodo.8383171, 13, 340, 348, 2019.

[71] G. Holland, A revised hurricane pressure-wind model. Mon. Weather Rev. 136, 3432–3445 (2008).

[72] K. Emanuel, R. Rotunno, Self-stratification of tropical cyclone outflow. Part I: Implications for storm structure. J. Atmos. Sci. 68, 2236–2249 (2011).

[73] D. G. Marks, The Beta and advection model for hurricane track forecasting (1992); https://repository.library.noaa.gov/view/noaa/7184.

[74] H. Hersbach, B. Bell, P. Berrisford, A. Horányi, J. M. Sabater, J. Nicolas, R. Radu, D. Schepers, A. Simmons, C. Soci, D. Dee, Global reanalysis: Goodbye ERA-Interim, hello ERA5. ECMWF Newsletter 159, 17–24 (2019).

[75] T. R. Knutson, J. J. Sirutis, M. Zhao, R. E. Tuleya, M. Bender, G. A. Vecchi, G. Villarini, D. Chavas, Global projections of intense tropical cyclone activity for the late twenty-first century from dynamical downscaling of CMIP5/RCP4.5 scenarios. J. Clim. 28, 7203–7224 (2015).

[76] A. H. Sobel, C. Y. Lee, S. J. Camargo, K. T. Mandli, K. A. Emanuel, P. Mukhopadhyay, M. Mahakur, Tropical cyclone hazard to mumbai in the recent historical climate. Mon. Weather Rev. 147, 2355–2366 (2019).

[77] IIASA, RCP Database (Version 2.0.5) (2009); https://tntcat.iiasa.ac.at/RcpDb/, Accessed on 2023-09-18.

[78] S. Eberenz, D. Stocker, T. Röösli, D. N. Bresch, Asset exposure data for global physical risk assessment. Earth Syst. Sci. Data 12, 817–833 (2020).

[79] J. Herman, W. Usher, SALib: An open-source python library for sensitivity analysis. J. Open Source Softw. 2, 97 (2017).

[80] N. Bloemendaal, I. I. Haigh, H. H. de Moel, S. Muis, R. R. Haarsma, J. J. Aerts, STORM IBTrACS present climate synthetic tropical cyclone tracks. 4TU.Centre for Research Data (2020); https://data.4tu.nl/articles/_/12706085/2, Accessed on 2024-02-29.

[81] N. Bloemendaal, H. H. de Moel, A. B. Martinez, S. S. Muis, I. I. Haigh, K. van der Wiel,R. R. Haarsma, P. P. Ward, M. Roberts, J. Dullaart, STORM Climate Change synthetic tropical cyclone tracks. 4TU.ResearchData (2023); https://data.4tu.nl/datasets/98900e17-8e01-4d70-b3b6-ca1a1da2f194/2, Accessed on 2024-02-29.

[82] S. Meiler, simonameiler/TC_future_risk_uncertainty_multi-model (2024); https://github.com/simonameiler/TC_future_risk_uncertainty_multi-model, Accessed on 2024-02-29.

[83] M. K. Tippett, S. J. Camargo, A. H. Sobel, A poisson regression index for tropical cyclone genesis and the role of large-scale vorticity in genesis. J. Clim. 24, 2335–2357 (2011).

[84] S. J. Camargo, M. K. Tippett, A. H. Sobel, G. A. Vecchi, M. Zhao, Testing the performance of tropical cyclone genesis indices in future climates using the HiRAM model. J. Clim. 27, 9171–9196 (2014).

[85] K. Emanuel, Response of global tropical cyclone activity to increasing CO_2_: Results from downscaling CMIP6 models. J. Clim. 34, 57–70 (2021).

[86] G. Danabasoglu, J.-F. Lamarque, J. Bacmeister, D. A. Bailey, A. K. DuVivier, J. Edwards, L. K. Emmons, J. Fasullo, R. Garcia, A. Gettelman, C. Hannay, M. M. Holland, W. G. Large, P. H. Lauritzen, D. M. Lawrence, J. T. M. Lenaerts, K. Lindsay, W. H. Lipscomb, M. J. Mills, R. Neale, K. W. Oleson, B. Otto-Bliesner, A. S. Phillips, W. Sacks, S. Tilmes, L. van Kampenhout, M. Vertenstein, A. Bertini, J. Dennis, C. Deser, C. Fischer, B. Fox-Kemper, J. E. Kay, D. Kinnison, P. J. Kushner, V. E. Larson, M. C. Long, S. Mickelson, J. K. Moore, E. Nienhouse, L. Polvani, P. J. Rasch, W. G. Strand, The community Earth system model version 2 (CESM2). J. Adv. Model. Earth Syst. 12, e2019MS001916 (2020).

[87] A. Voldoire, D. Saint-Martin, S. Sénési, B. Decharme, A. Alias, M. Chevallier, J. Colin, J.-F. Guérémy, M. Michou, M.-P. Moine, P. Nabat, R. Roehrig, D. Salas y Mélia, D. S. y Mélia, R. Séférian, S. Valcke, I. Beau, S. Belamari, S. Berthet, C. Cassou, J. Cattiaux, J. Deshayes, H. Douville, C. Ethé, L. Franchistéguy, O. Geoffroy, C. Lévy, G. Madec, Y. Meurdesoif, R. Msadek, A. Ribes, E. Sanchez-Gomez, L. Terray, R. Waldman, Evaluation of CMIP6 DECK experiments with CNRM-CM6-1. J. Adv. Model. Earth Syst. 11, 2177–2213 (2019).

[88] EC Earth Consortium, EC-Earth-Consortium EC-Earth3 model output prepared for CMIP6 ScenarioMIP ssp245 (2019); 10.22033/ESGF/CMIP6.4880, Accessed on 2023-02-16.

[89] L. Li, CAS FGOALS-g3 model output prepared for CMIP6 ScenarioMIP ssp245 (2019); 10.22033/ESGF/CMIP6.3469, Accessed on 2023-02-16.

[90] F. Hourdin, C. Rio, J.-Y. Grandpeix, J.-B. Madeleine, F. Cheruy, N. Rochetin, A. Jam, I. Musat, A. Idelkadi, L. Fairhead, M.-A. Foujols, L. Mellul, A.-K. Traore, J.-L. Dufresne, O. Boucher, M.-P. Lefebvre, E. Millour, E. Vignon, J. Jouhaud, F. B. Diallo, F. Lott, G. Gastineau, A. Caubel, Y. Meurdesoif, J. Ghattas, LMDZ6A: The atmospheric component of the IPSL climate model with improved and better tuned physics. J. Adv. Model. Earth Syst. 12, e2019MS001892 (2020).

[91] H. Tatebe, T. Ogura, T. Nitta, Y. Komuro, K. Ogochi, T. Takemura, K. Sudo, M. Sekiguchi, M. Abe, F. Saito, M. Chikira, S. Watanabe, M. Mori, N. Hirota, Y. Kawatani, T. Mochizuki, K. Yoshimura, K. Takata, R. O’ishi, D. Yamazaki, T. Suzuki, M. Kurogi, T. Kataoka, M. Watanabe, M. Kimoto, Description and basic evaluation of simulated mean state, internal variability, and climate sensitivity in MIROC6. Geosci. Model Dev. 12, 2727–2765 (2019).

[92] W. A. Müller, J. H. Jungclaus, T. Mauritsen, J. Baehr, M. Bittner, R. Budich, F. Bunzel, M. Esch, R. Ghosh, H. Haak, T. Ilyina, T. Kleine, L. Kornblueh, H. Li, K. Modali, D. Notz, H. Pohlmann, E. Roeckner, I. Stemmler, F. Tian, J. Marotzke, A higher-resolution version of the Max Planck Institute Earth System Model (MPI-ESM1.2-HR). J. Adv. Model. Earth Syst. 10, 1383–1413 (2018).

[93] S. Yukimoto, T. Koshiro, H. Kawai, N. Oshima, K. Yoshida, S. Urakawa, H. Tsujino, M. Deushi, T. Tanaka, M. Hosaka, H. Yoshimura, E. Shindo, R. Mizuta, M. Ishii, A. Obata, Y. Adachi, MRI MRI-ESM2.0 model output prepared for CMIP6 ScenarioMIP ssp245 (2019); 10.22033/ESGF/CMIP6.6910, Accessed on 2023-02-16.

[94] A. A. Sellar, J. Walton, C. G. Jones, R. Wood, N. L. Abraham, M. Andrejczuk, M. B. Andrews, T. Andrews, A. T. Archibald, L. de Mora, H. Dyson, M. Elkington, R. Ellis, P. Florek, P. Good, L. Gohar, S. Haddad, S. C. Hardiman, E. Hogan, A. Iwi, C. D. Jones, B. Johnson, D. I. Kelley, J. Kettleborough, J. R. Knight, M. O. Köhler, T. Kuhlbrodt, S. Liddicoat, I. Linova-Pavlova, M. S. Mizielinski, O. Morgenstern, J. Mulcahy, E. Neininger, F. M. O’Connor, R. Petrie, J. Ridley, J.-C. Rioual, M. Roberts, E. Robertson, S. Rumbold, J. Seddon, H. Shepherd, S. Shim, A. Stephens, J. C. Teixiera, Y. Tang, J. Williams, A. Wiltshire, P. T. Griffiths, Implementation of U.K. Earth system models for CMIP6. J. Adv. Model. Earth Syst. 12, e2019MS001946 (2020).

[95] Z. Hausfather, K. Marvel, G. A. Schmidt, J. W. Nielsen-Gammon, M. Zelinka, Climate simulations: Recognize the ‘hot model’ problem. Nature 605, 26–29 (2022). 35508771 10.1038/d41586-022-01192-2

