# Supplementary material for: Navigating and attributing uncertainty in future tropical cyclone risk estimates
Source: Sci Adv. 2025 Apr 18;11(16):eadn4607. doi: 10.1126/sciadv.adn4607 (PMC13108827; doi:10.1126/sciadv.adn4607)
Supplement: Supplementary file 1 — Supplementary Discussion Figs. S1 to S12 Tables S1 to S5 Legend for table S6 References [file sciadv.adn4607_sm.pdf]

Science Advances
AAAS

Supplementary Materials for

Navigating and attributing uncertainty in future tropical cyclone risk estimates

Simona Meiler *et al.*

Corresponding author: Simona Meiler, simona@simonameiler.ch

Sci. Adv. **11**, eadn4607 (2025)
DOI: 10.1126/sciadv.adn4607

The PDF file includes:

Supplementary Discussion
Figs. S1 to S12
Tables S1 to S5
Legend for table S6
References

Other Supplementary Material for this manuscript includes the following:

Table S6

Supplementary Discussion

We investigate and discuss the role of the two distinctly different moisture variables used in the tropical cyclone genesis index (TCGI) component of CHAZ, which modulate the resulting CHAZ hazard frequency (38). Specifically, event sets generated using column-integral relative humidity (CRH) (83) as a moisture variable show an increase in TC frequencies in a warming climate, whereas those based on saturation deficit (SD) (84) indicate a decrease (fig. S10). Despite this distinct divergence in TC frequencies, similar variations are not observed in the TC risk changes when using CHAZ (fig. S8 and fig. S9). Furthermore, the sensitivity indices for the TCGI variable are not the highest (Fig. 4 (main text)). On the other hand, events generated using both CRH and SD as moisture variables offer comparable TC risk change estimates, although CRH-TCGI-based hazard sets generally exhibit higher maxima (fig. S8 and fig. S9).

This smaller impact of TCGI on risk estimates, in contrast to its evident role in hazard frequency, can be attributed to CHAZ hazard intensity. In this aspect, the choice of GCM exerts a more substantial influence than the TCGI moisture variable (fig. S11). Given these insights, we argue that TCGI selection may be of secondary importance in a risk modeling context, especially when socio-economic and exposure-related uncertainties are more pronounced. Nonetheless, using both TCGI versions is advisable to avert possible blind spots in representing future TC risks. Regarding model refinement, both the choice of TCGI and GCM remain critical aspects of epistemic uncertainty that warrant further investigation.

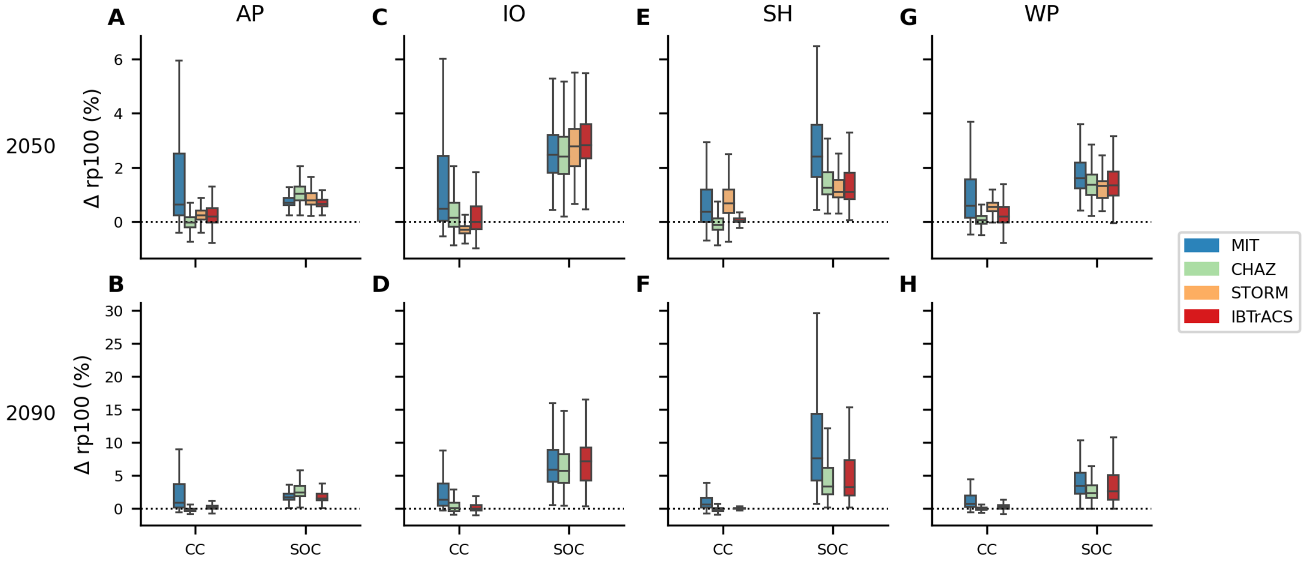

Fig. S1: Drivers of future tropical cyclone risk change - 100-yr event. Relative change in 100-yr event (rp100) by 2050 (top row; A, C, E, G) and 2090 (bottom row; B, D, F, H) due to climate change (CC) and socio-economic development (SOC) with respect to the historical baseline. The relative change rp100 is reported for the four study regions (A, B: North Atlantic/Eastern Pacific (AP), C, D: North Indian Ocean (IO), E, F: Southern Hemisphere (SH), and G, H: North Western Pacific (WP)). Boxplots are shown for the four models MIT (blue), CHAZ (green), STORM (orange), IBTrACS_p (red) and display the interquartile range (IQR) for the uncertainty over all input factors (see Methods), while the whiskers extend to 1.5 times the IQR. More extreme points (outliers) are not shown. Note that STORM results are only available for 2050.

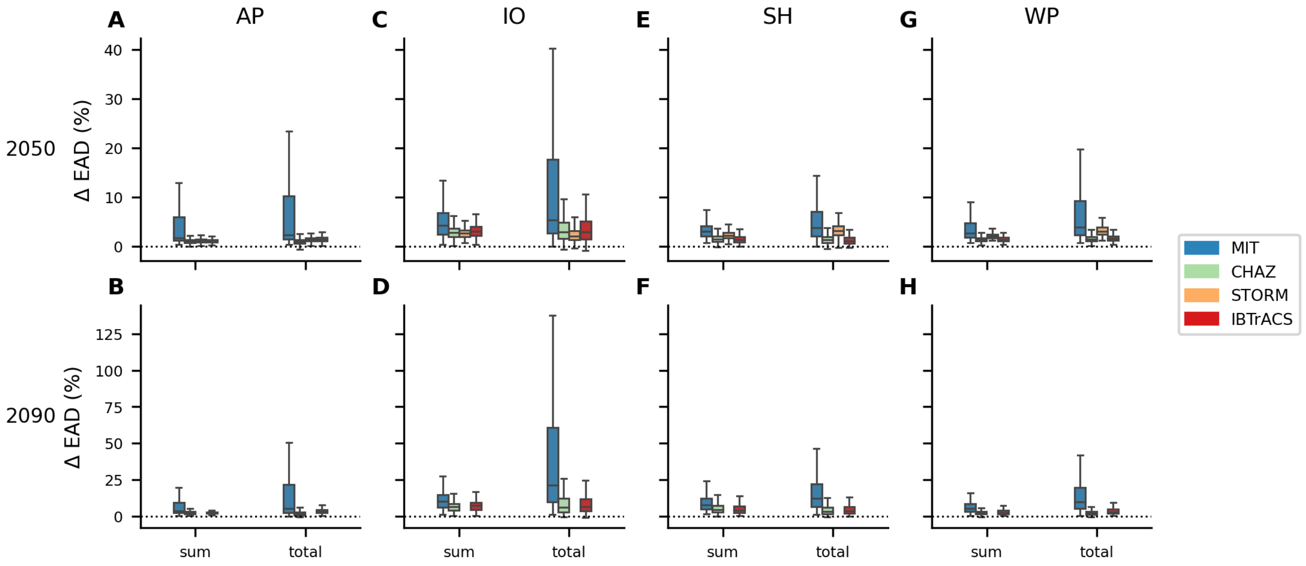

Fig. S2: Total future tropical cyclone risk change - EAD. Relative change in expected annual damage (EAD) by 2050 (top row; A, C, E, G) and 2090 (bottom row; B, D, F, H) due to the product of CC and SOC calculated from the sum of their log values (sum) and both drivers interacting (total) with respect to the historical baseline. The relative change EAD is reported for the four study regions (A, B: North Atlantic/Eastern Pacific (AP), C, D: North Indian Ocean (IO), E, F: Southern Hemisphere (SH), and G, H: North Western Pacific (WP)). Boxplots are shown for the four models MIT (blue), CHAZ (green), STORM (orange), IBTrACS_p (red) and display the interquartile range (IQR) for the uncertainty over all input factors (see Methods), while the whiskers extend to 1.5 times the IQR. More extreme points (outliers) are not shown. Note that STORM results are only available for 2050.

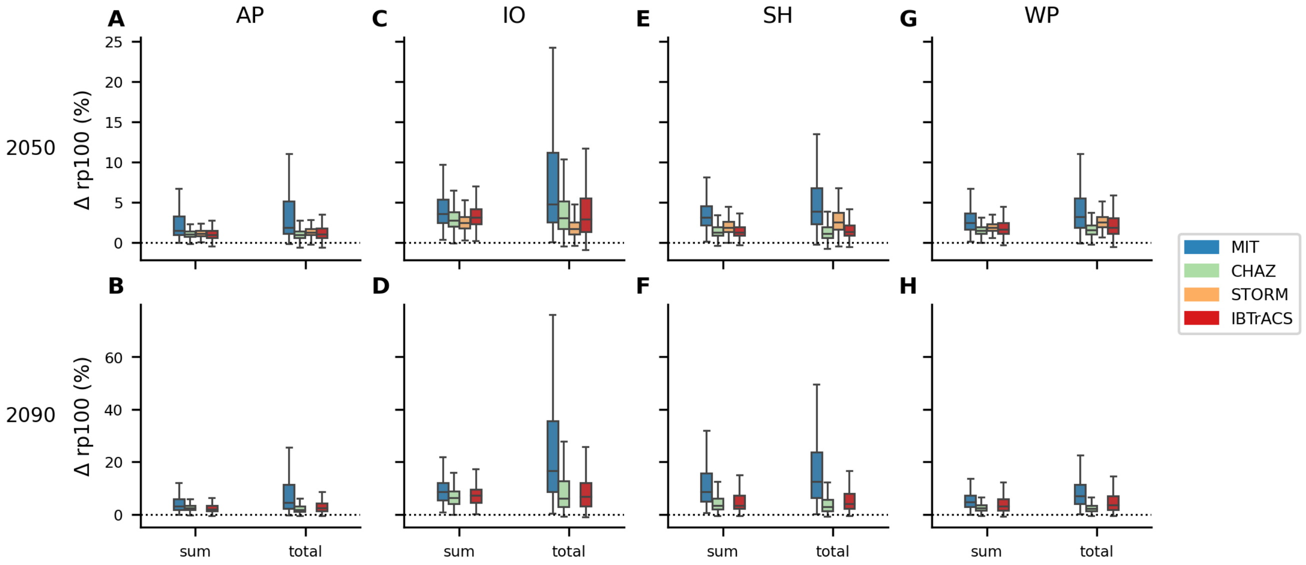

Fig. S3: Total future tropical cyclone risk change - 100-yr event. Relative change in 100-yr event (rp100) by 2050 (top row; A, C, E, G) and 2090 (bottom row; B, D, F, H) the product of CC and SOC calculated from the sum of their log values (sum) and both drivers interacting (total) with respect to the historical baseline. The relative change rp100 is reported for the four study regions (A, B: North Atlantic/Eastern Pacific (AP), C, D: North Indian Ocean (IO), E, F: Southern Hemisphere (SH), and G, H: North Western Pacific (WP)). Boxplots are shown for the four models MIT (blue), CHAZ (green), STORM (orange), IBTrACS_p (red) and display the interquartile range (IQR) for the uncertainty over all input factors (see Methods), while the whiskers extend to 1.5 times the IQR. More extreme points (outliers) are not shown. Note that STORM results are only available for 2050.

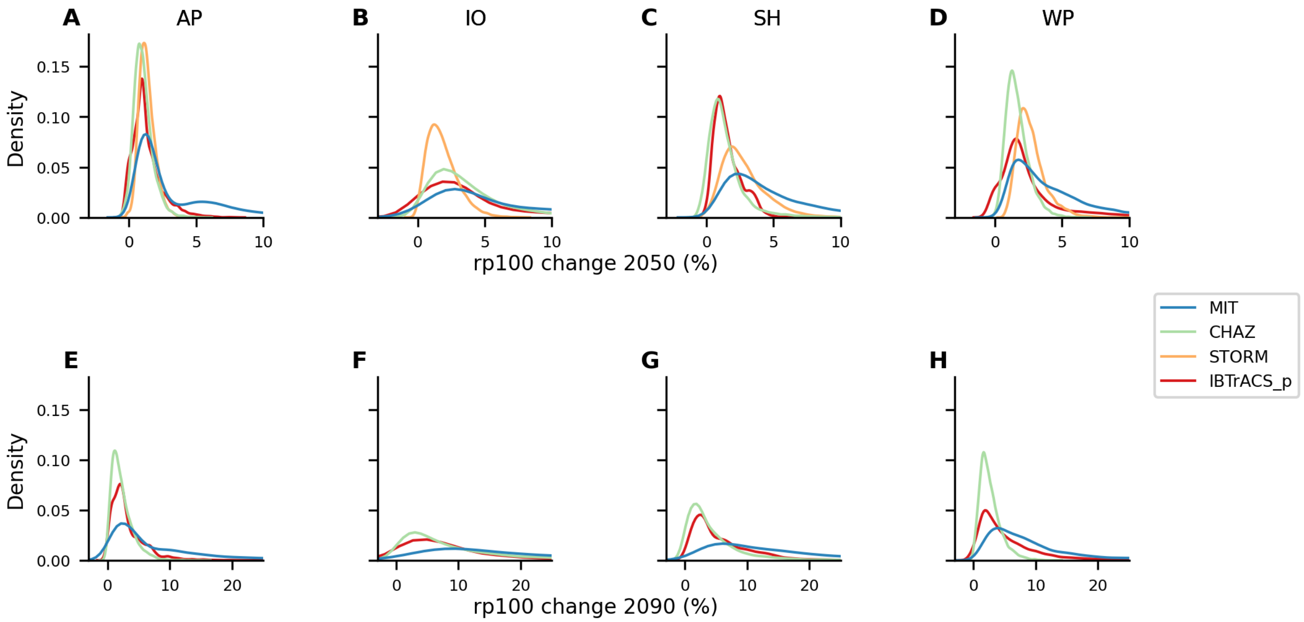

Fig. S4: Uncertainty distribution of TC risk change. Kernel density estimation plots showcasing the uncertainty distribution of estimated relative change in 100-yr event (rp100) across study regions (A, E: North Atlantic/Eastern Pacific (AP), B, F: North Indian Ocean (IO), C, G: Southern Hemisphere (SH), and D, H: North Western Pacific (WP)) for the years 2050 (top row: A, B, C, D) and 2090 (bottom row: E, F, G, H). Each subplot represents a specific region and year combination, with different models (MIT, CHAZ, STORM, IBTrACS_p) depicted in distinct colors. Note, the model STORM only provides data for 2050. Each plot shows a normalized probability distribution with an integral sum of 1. The x-axis is truncated in some figures, potentially influencing the interpretation of distribution tails, particularly for the MIT hazard-based results.

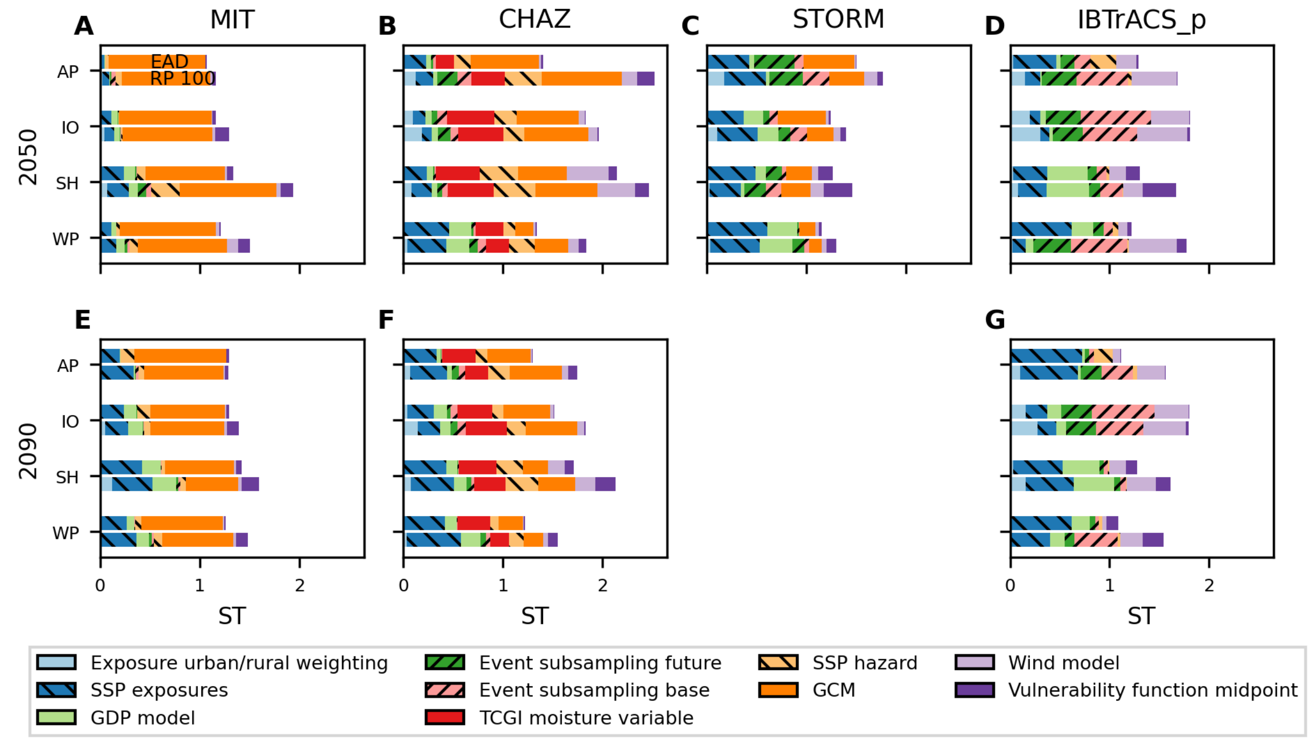

Fig. S5: Total-order sensitivity indices of future TC risk change. Total-order Sobol sensitivity indices for future (2050: A, B, C, D; 2090: E, F, G) TC risk change calculated with the four models (MIT: A, E; CHAZ: B, F; STORM: C; IBTrACS_p: D, G), expressed as %-change in expected annual damage (EAD; upper bar) and 100-yr event values (RP 100; lower bar) over the four study regions (North Atlantic/Eastern Pacific (AP), North Indian Ocean (IO), Southern Hemisphere (SH), and North Western Pacific (WP)) and all input factors (different colors). Input factors that primarily constitute aleatory uncertainty are shown in forward-slanting hatching; scenario uncertainty in backward-slanting hatching. *Vulnerability func. midp.* describes the impact function; *Wind model*; *GCM*, *SSP hazard*, *TCGI moisture variable*, *Event subsampling base*, *Event subsampling future* pertain to the hazard component; *GDP model*; *SSP exposure*, *Exposure urban/rural weighting* relate to the exposure. Note that STORM results are only available for 2050.

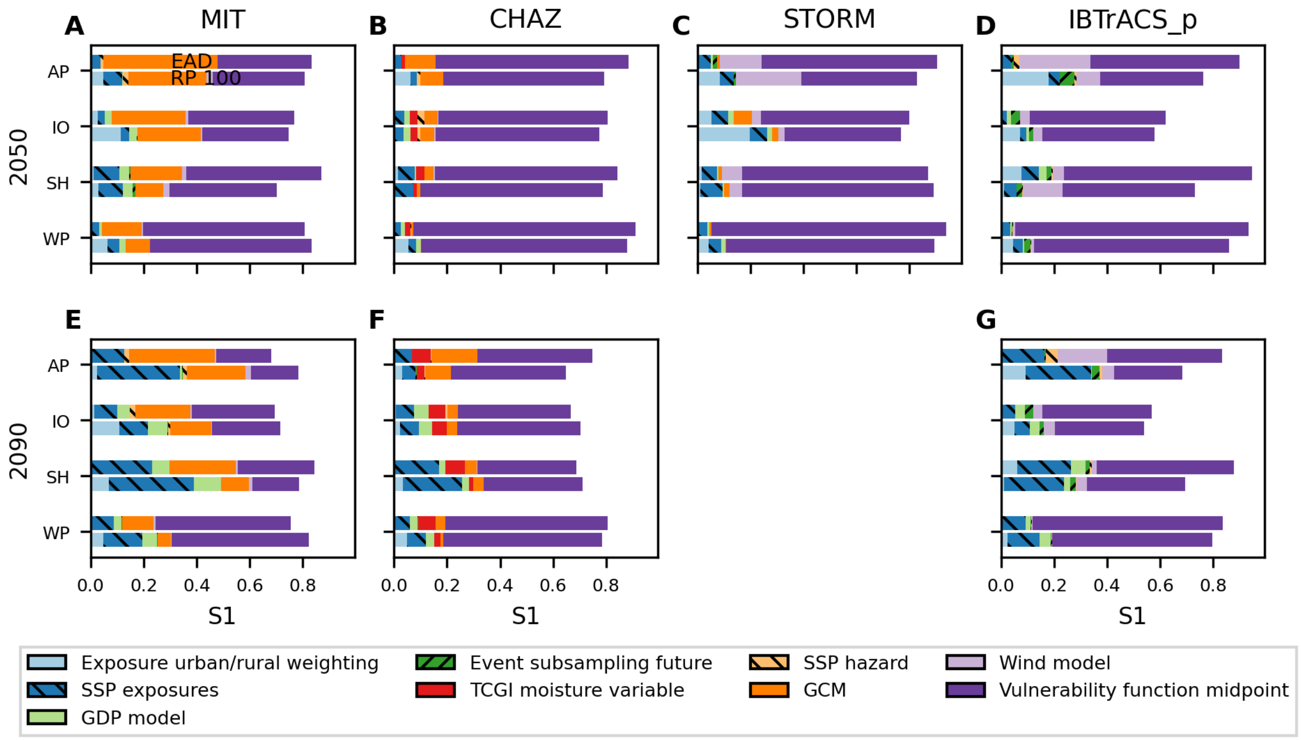

Fig. S6: First-order sensitivity indices of absolute future TC risk. First-order Sobol sensitivity indices for future (2050: A, B, C, D; 2090: E, F, G) TC risk calculated with the four models (MIT: A, E; CHAZ: B, F; STORM: C; IBTrACS_p: D, G), expressed as absolute (calculated in USD) expected annual damage (EAD; upper bar) and 100-yr event values (RP 100; lower bar) over the four study regions (North Atlantic/Eastern Pacific (AP), North Indian Ocean (IO), Southern Hemisphere (SH), and North Western Pacific (WP) and all input factors (different colors). Input factors that primarily constitute aleatory uncertainty are shown in forward-slanting hatching; scenario uncertainty in backward-slanting hatching. *Vulnerability func. midp.* describes the impact function; *Wind model*; *GCM*, *SSP hazard*, *TCGI moisture variable*, *Event subsampling base*, *Event subsampling future* pertain to the hazard component; *GDP model*; *SSP exposure*, *Exposure urban/rural weighting* relate to the exposure. Note that STORM results are only available for 2050.

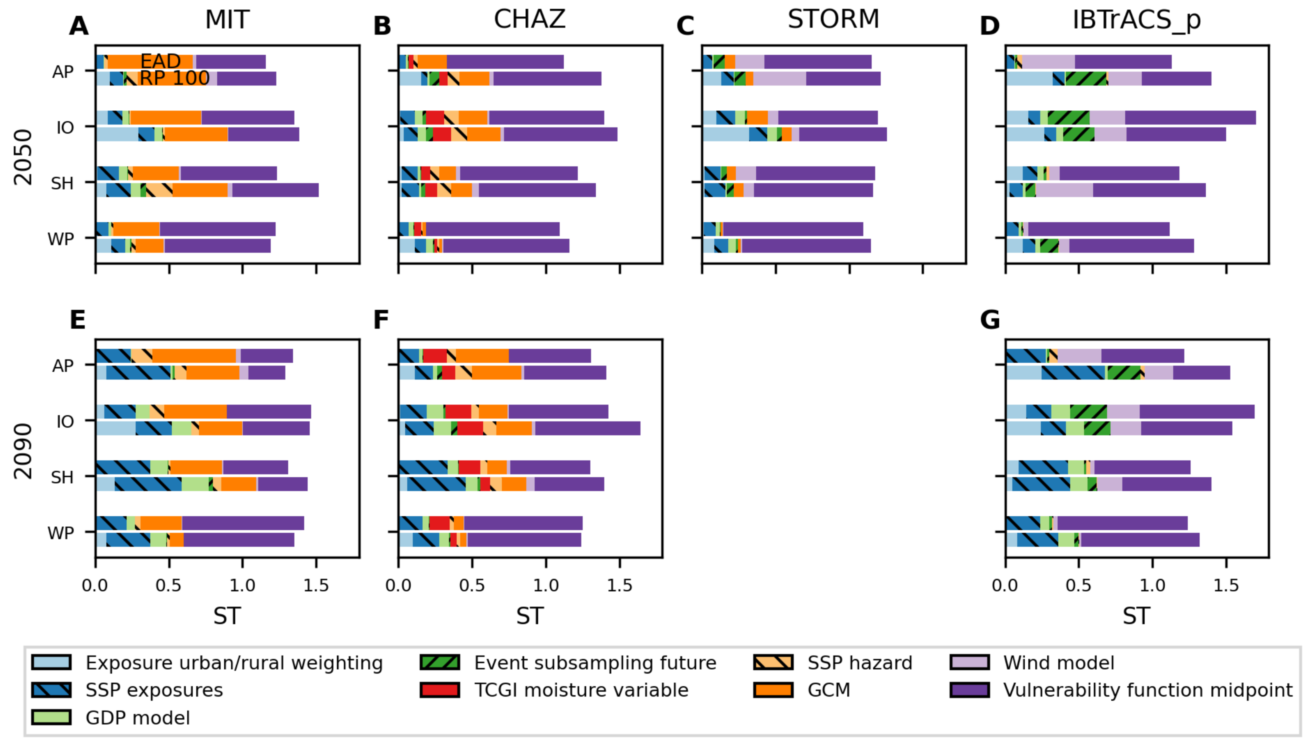

Fig. S7: Total-order sensitivity indices of absolute future TC risk. Total-order Sobol sensitivity indices for future (2050: A, B, C, D; 2090: E, F, G) TC risk calculated with the four models (MIT: A, E; CHAZ: B, F; STORM: C; IBTrACS_p: D, G), expressed as absolute (calculated in USD) expected annual damage (EAD; upper bar) and 100-yr event values (RP 100; lower bar) over the four study regions (North Atlantic/Eastern Pacific (AP), North Indian Ocean (IO), Southern Hemisphere (SH), and North Western Pacific (WP) and all input factors (different colors). Input factors that primarily constitute aleatory uncertainty are shown in forward-slanting hatching; scenario uncertainty in backward-slanting hatching. *Vulnerability func. midp.* describes the impact function; *Wind model*; *GCM*, *SSP hazard*, *TCGI moisture variable*, *Event subsampling base*, *Event subsampling future* pertain to the hazard component; *GDP model*; *SSP exposure*, *Exposure urban/rural weighting* relate to the exposure. Note that STORM results are only available for 2050.

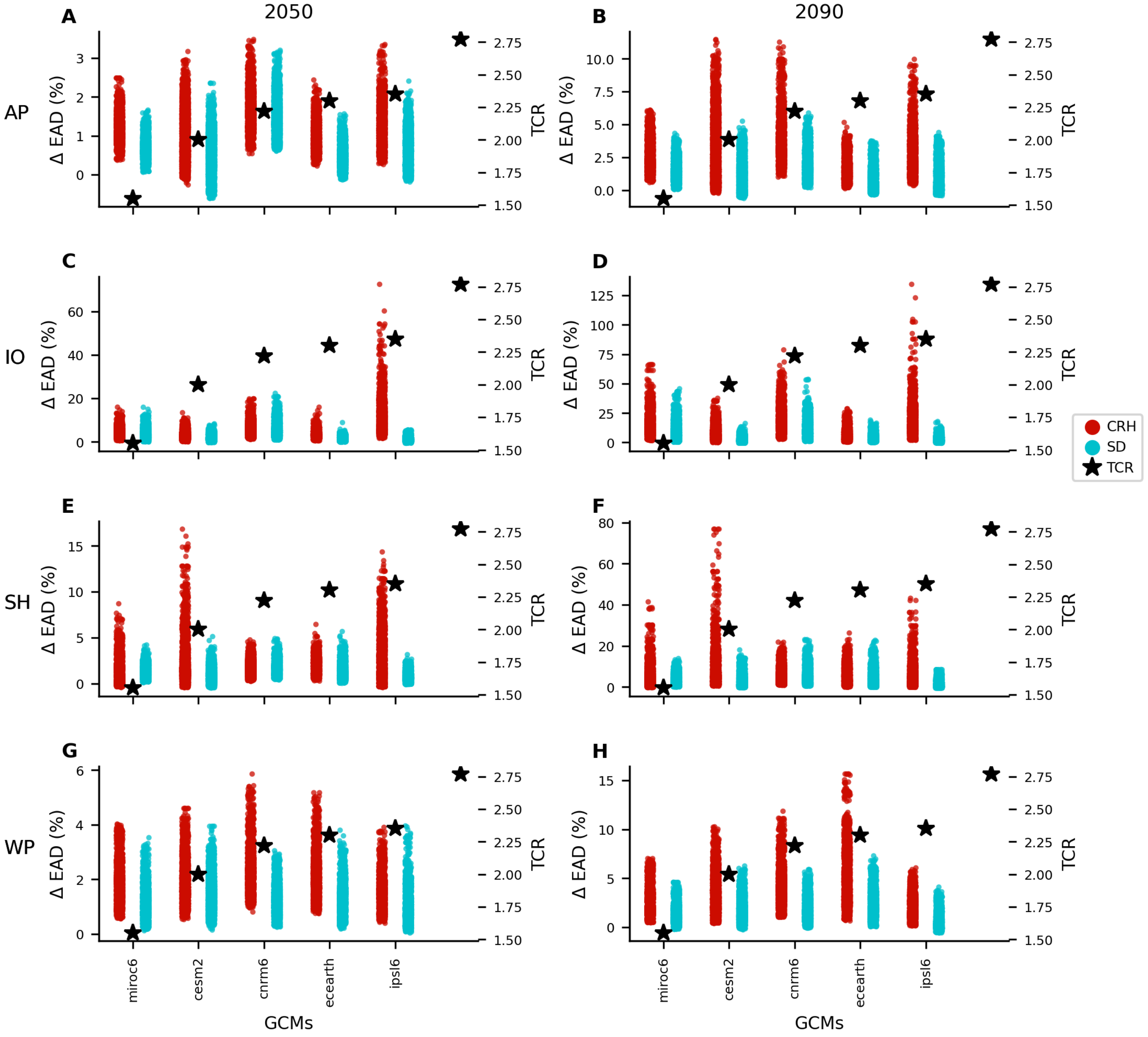

Fig. S8: EAD change in CHAZ apportioned to GCMs and TCGI variables. Model simulations of the expected annual damage (EAD) change by 2050 (A, C, E, G) and 2090 (B, D, F, H) attributed to the six GCMs and two moisture variables used in the TCGI underlying the CHAZ TC hazard sets. GCMs are ordered by increasing transient climate response (TCR) values (table S5), which are shown as black stars on a secondary y-axis. Results are shown over the four study regions North Atlantic/Eastern Pacific (AP: A, B), North Indian Ocean (IO: C, D), Southern Hemisphere (SH: E, F), and North Western Pacific (WP: G, H).

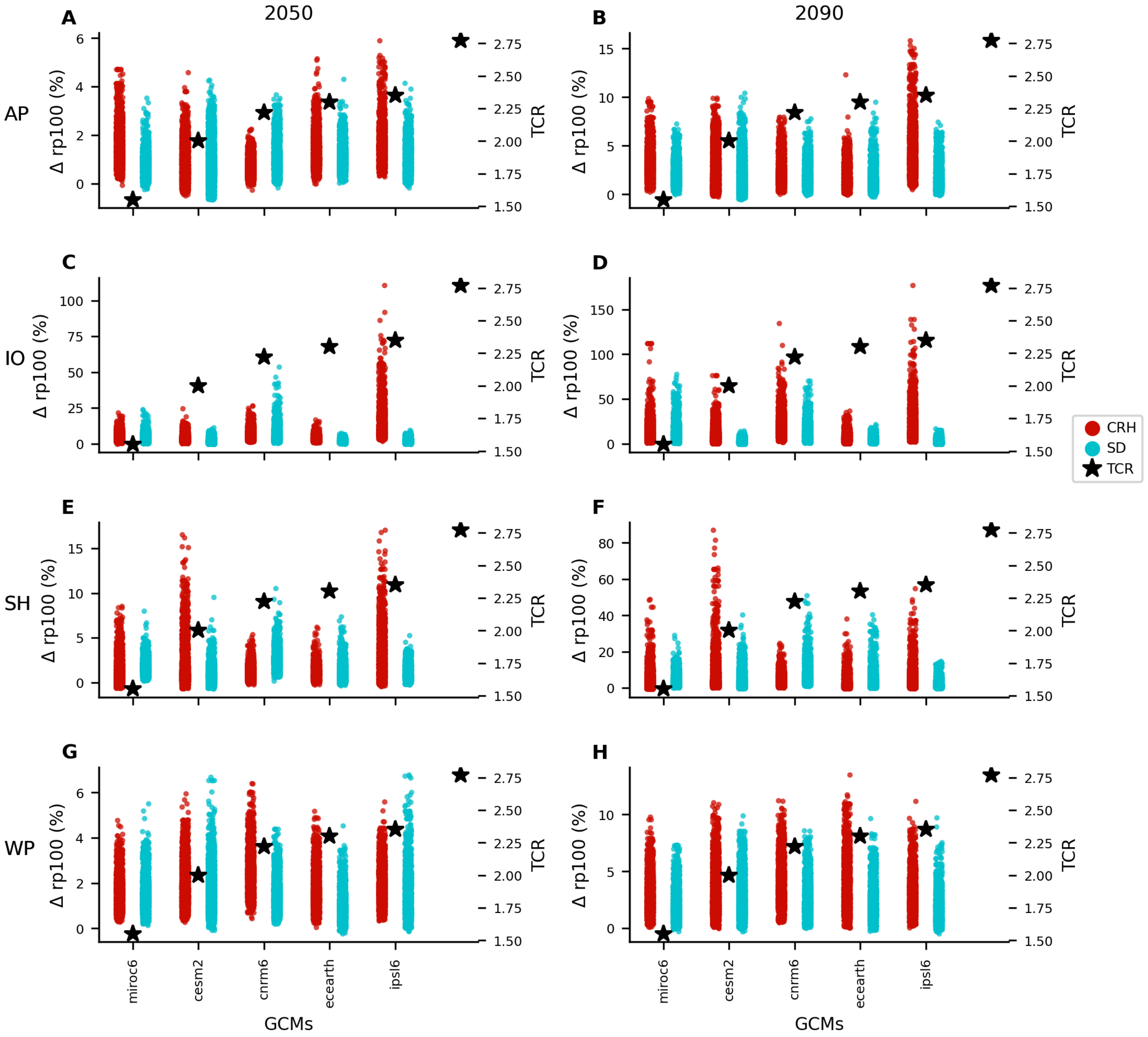

Fig. S9: RP100 change in CHAZ apportioned to GCMs and TCGI variables. Model simulations of the 100-yr event (rp100) change by 2050 (A, C, E, G) and 2090 (B, D, F, H) attributed to the six GCMs and two moisture variables used in the TCGI underlying the CHAZ TC hazard sets. GCMs are ordered by increasing transient climate response (TCR) values (table S5), which are shown as black stars on a secondary y-axis. Results are shown over the four study regions North Atlantic/Eastern Pacific (AP: A, B), North Indian Ocean (IO: C, D), Southern Hemisphere (SH: E, F), and North Western Pacific (WP: G, H).

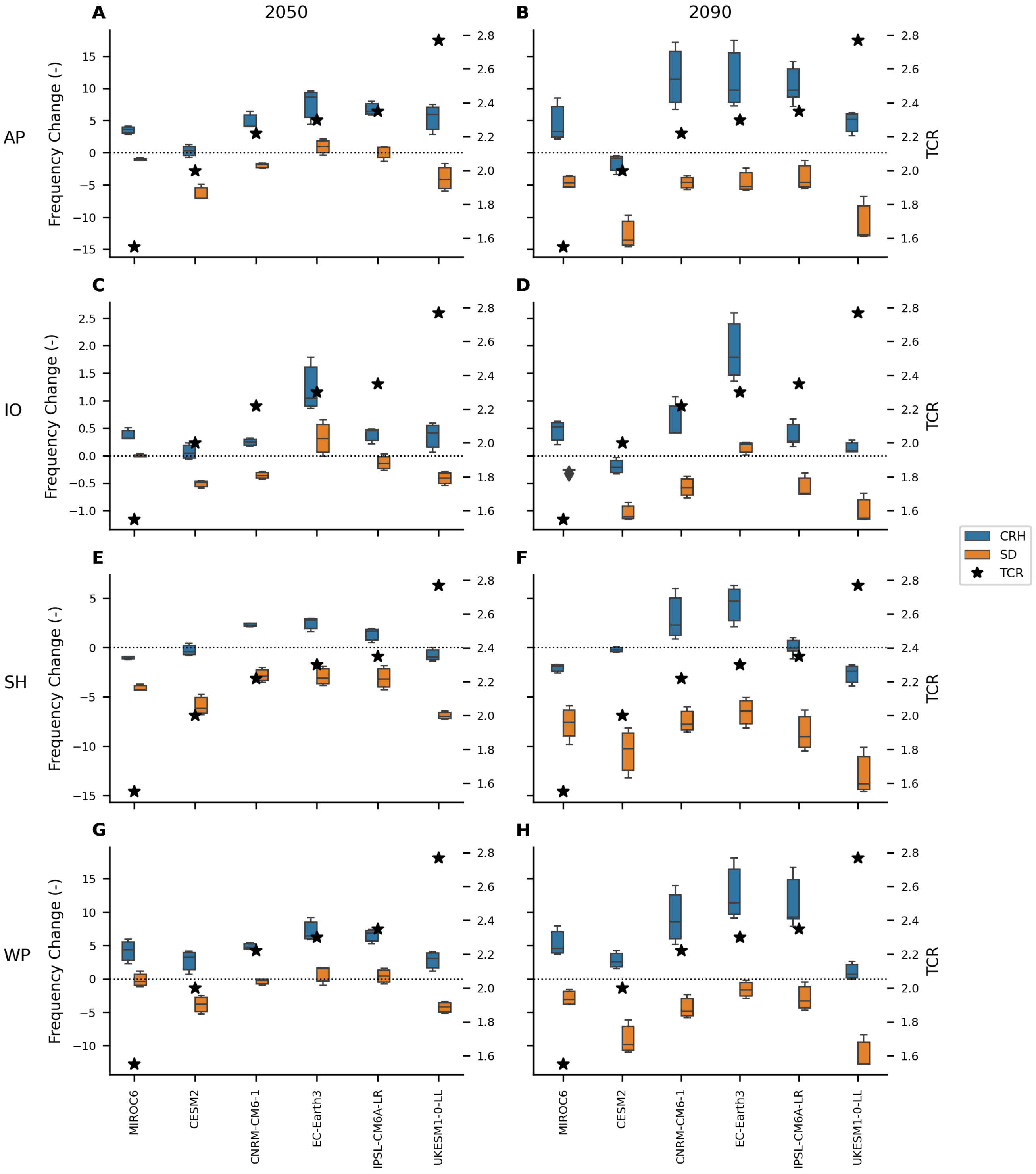

Fig. S10: Frequency changes in CHAZ hazard sets. CHAZ hazard frequency change values for event sets of the six different GCMs, separated by the two TCGI moisture variables (CRH, SD) and shown for two future time periods (2050: A, C, E, F; 2090: B, D, F, H) and four study regions North Atlantic/Eastern Pacific (AP: A, B), North Indian Ocean (IO: C, D), Southern Hemisphere (SH: E, F), and North Western Pacific (WP: G, H). Frequency change values were calculated relative to the historical period and analyzed for the full event set, hence not limited to land-influencing storms. Additionally, transient climate response (TCR) values for the six GCMs are shown on a secondary y-axis (see table S5).

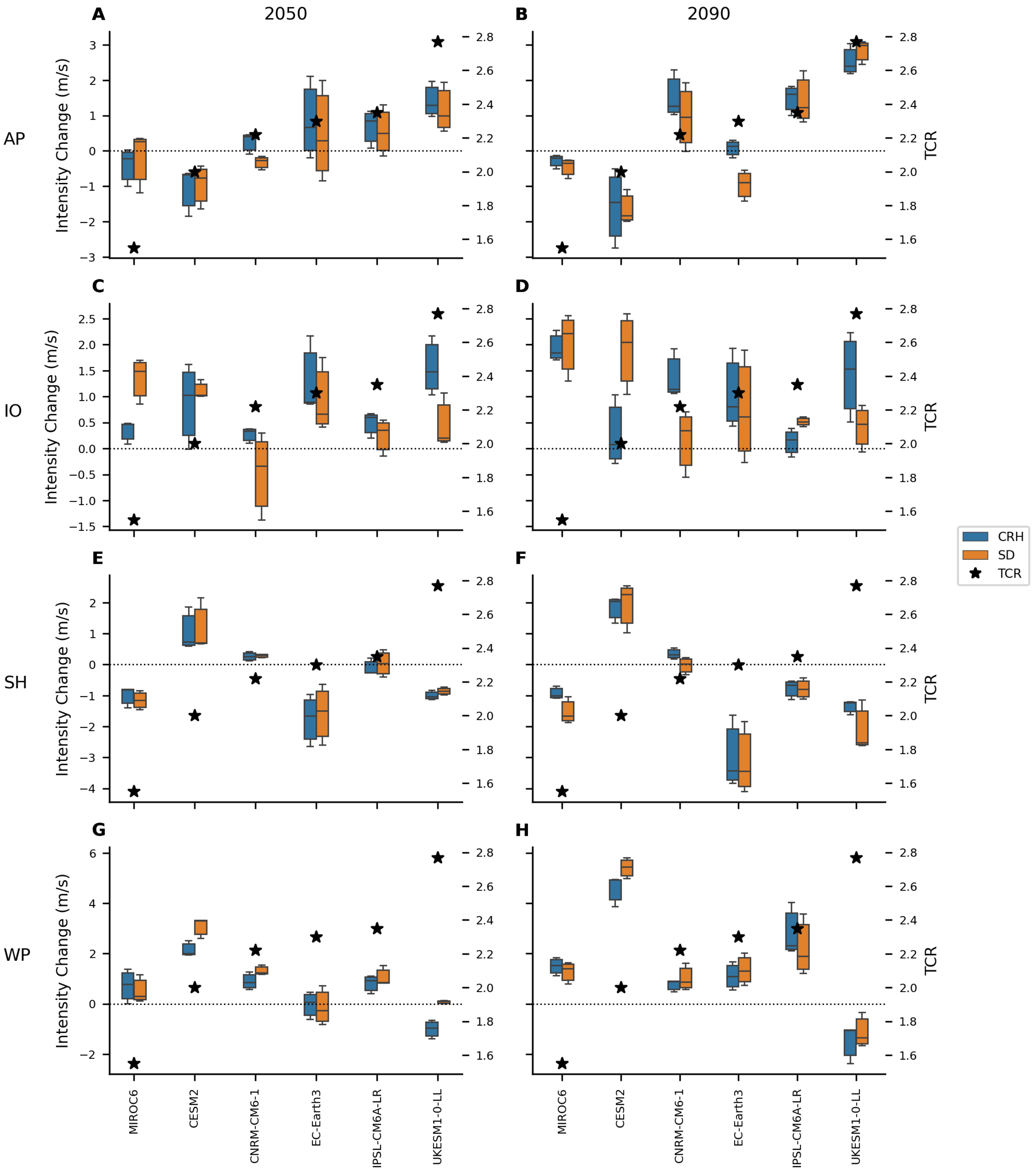

Fig. S11: Intensity changes in CHAZ hazard sets. CHAZ hazard intensity change values for event sets of the six different GCMs, separated by the two TCGI moisture variables (CRH, SD) and shown for two future time periods (2050: A, C, E, F; 2090: B, D, F, H) and four study regions North Atlantic/Eastern Pacific (AP: A, B), North Indian Ocean (IO: C, D), Southern Hemisphere (SH: E, F), and North Western Pacific (WP: G, H). Intensity change values were derived for both wind models used in the hazard generation (71, 85). Intensity changes are calculated as the mean over the maximum sustained wind speeds of all TCs in the future event sets minus the equivalent of the historical period. Note, we analyze the full event set and do not limit the analysis to land-influencing storms. Additionally, transient climate response (TCR) values for the six GCMs are shown on a secondary y-axis (see table S5).

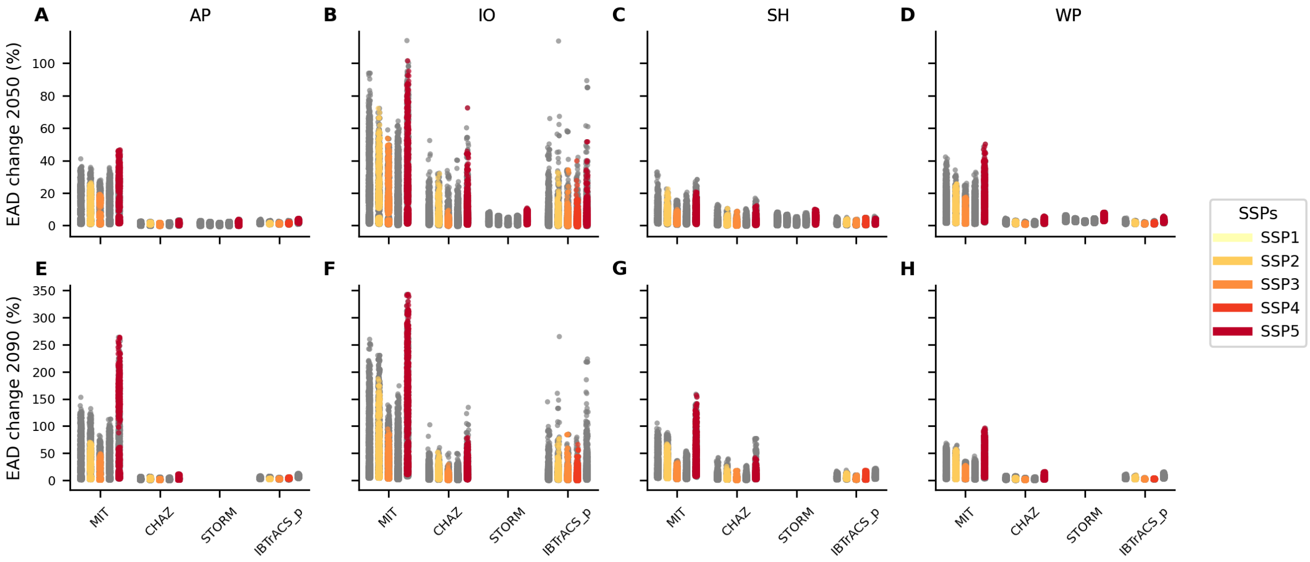

Fig. S12: Uncertainty distribution of TC risk change by TC model and SSP scenario. Model simulations of the expected annual damage (EAD) change by 2050 (A, B, C, D) and 2090 (E, F, G, H) attributed to the four TC models (MIT, CHAZ, STORM, IBTrACS_p). Results are partitioned by SSP scenario, with uncertainty outputs categorized by the input factor *SSP exposure* (as defined in the uncertainty and sensitivity analysis; see table S1). Matching emission scenarios (*SSP hazard*) are displayed in color according to availability for each TC model. Grey dots indicate results from scenarios where the SSP exposure and SSP hazard combinations do not align, following conventional definitions. Note that the model STORM only provides data for 2050. Results are shown over the four study regions North Atlantic/Eastern Pacific (AP: A, B), North Indian Ocean (IO: C, D), Southern Hemisphere (SH: E, F), and North Western Pacific (WP: G, H).

region	year	model	Δ EAD (%)	Δ rp100 (%)
AP	2050	MIT	1.63	1.23
		CHAZ	0.80	0.76
		STORM	1.27	1.13
		IBTrACS_p	1.18	0.97
	2090	MIT	3.39	2.41
		CHAZ	1.32	1.16
		STORM	N/A	N/A
		IBTrACS_p	2.40	2.06
IO	2050	MIT	2.76	2.75
		CHAZ	1.80	1.97
		STORM	1.45	1.23
		IBTrACS_p	1.74	2.19
	2090	MIT	10.00	9.04
		CHAZ	2.84	2.95
		STORM	N/A	N/A
		IBTrACS_p	4.03	4.20
SH	2050	MIT	1.84	2.34
		CHAZ	1.03	0.86
		STORM	3.00	1.91
		IBTrACS_p	0.73	0.99
	2090	MIT	6.06	6.16
		CHAZ	1.58	1.71
		STORM	N/A	N/A
		IBTrACS_p	1.81	2.48
WP	2050	MIT	2.36	1.74
		CHAZ	1.29	1.27
		STORM	2.67	2.10
		IBTrACS_p	1.31	1.57
	2090	MIT	4.99	3.88
		CHAZ	1.35	1.65
		STORM	N/A	N/A
		IBTrACS_p	1.91	1.92

Table S1: Maximum kernel density of TC risk change uncertainty distribution. Maximum kernel density estimation of TC risk change uncertainty distribution for estimated change in expected annual damage (EAD) and 100-yr event (rp100) across study regions (North Atlantic/Eastern Pacific (AP), North Indian Ocean (IO), Southern Hemisphere (SH), and North Western Pacific (WP)) for the years 2050 and 2090 and the four models (MIT, CHAZ, STORM, IBTrACS_p). The full uncertainty distribution is shown in Fig. 3 (main text) and fig. S4.

region	year	model	S1 EAD	S1 rp100	ST EAD	ST rp100
AP	2050	MIT	GCM	GCM	GCM	GCM
		CHAZ	GCM	GCM	GCM	GCM
		STORM	SSP exposure	SSP exposure	GCM	SSP exposure
		IBTrACS_p	SSP exposure	Event subsampling base	SSP exposure	Event subsampling base
	2090	MIT	GCM	GCM	GCM	GCM
		CHAZ	GCM	SSP exposure	GCM	GCM
		STORM	N/A	N/A	N/A	N/A
		IBTrACS_p	SSP exposure	SSP exposure	SSP exposure	SSP exposure
IO	2050	MIT	GCM	GCM	GCM	GCM
		CHAZ	GCM	GCM	GCM	GCM
		STORM	GCM	SSP exposure	GCM	SSP exposure
		IBTrACS_p	Event subsampling future	Event subsampling base	Event subsampling base	Event subsampling base
	2090	MIT	GCM	GCM	GCM	GCM
		CHAZ	GCM	TCGI moisture var.	GCM	GCM
		STORM	N/A	N/A	N/A	N/A
		IBTrACS_p	Event subsampling future	Event subsampling base	Event subsampling base	Event subsampling base
SH	2050	MIT	GCM	GCM	GCM	GCM
		CHAZ	SSP exposure	SSP exposure	GCM	GCM
		STORM	SSP exposure	SSP exposure	SSP exposure	Vulnerability func. midp.
		IBTrACS_p	SSP exposure	SSP exposure	GDP model	GDP model
	2090	MIT	GCM	SSP exposure	GCM	GCM
		CHAZ	SSP exposure	SSP exposure	SSP exposure	SSP exposure
		STORM	N/A	N/A	N/A	N/A
		IBTrACS_p	SSP exposure	SSP exposure	SSP exposure	SSP exposure
WP	2050	MIT	GCM	GCM	GCM	GCM
		CHAZ	SSP exposure	SSP exposure	SSP exposure	SSP exposure
		STORM	SSP exposure	SSP exposure	SSP exposure	SSP exposure
		IBTrACS_p	SSP exposure	Event subsampling base	SSP exposure	Event subsampling base
	2090	MIT	GCM	GCM	GCM	GCM
		CHAZ	SSP exposure	SSP exposure	SSP exposure	SSP exposure
		STORM	N/A	N/A	N/A	N/A
		IBTrACS_p	SSP exposure	SSP exposure	SSP exposure	Event subsampling base

Table S2: Largest sensitivity indices for future TC risk change estimates. Highest first- (S1) and total-order (ST) Sobol sensitivity indices for both risk change metrics (expected annual damage (EAD) and 100-yr event (rp100)), expressed as %-change in the four study regions (North Atlantic/Eastern Pacific (AP), North Indian Ocean (IO), Southern Hemisphere (SH), and North Western Pacific (WP) for both future periods (2050, 2090) and all four models (MIT, CHAZ, STORM, IBTrACS_p. Plots showing all sensitivity indices can be found in Fig. 4 (main text) and fig. S5.

region	year	model	S1 EAD	S1 rp100	ST EAD	ST rp100
AP	2050	MIT	GCM	GCM	GCM	GCM
		CHAZ	Vulnerability func. midp.	Vulnerability func. midp.	Vulnerability func. midp.	Vulnerability func. midp.
		STORM	Vulnerability func. midp.	Vulnerability func. midp.	Vulnerability func. midp.	Vulnerability func. midp.
		IBTrACS_p	Vulnerability func. midp.	Vulnerability func. midp.	Vulnerability func. midp.	Vulnerability func. midp.
	2090	MIT	GCM	SSP exposure	GCM	SSP exposure
		CHAZ	Vulnerability func. midp.	Vulnerability func. midp.	Vulnerability func. midp.	Vulnerability func. midp.
		STORM	N/A	N/A	N/A	N/A
		IBTrACS_p	Vulnerability func. midp.	Vulnerability func. midp.	Vulnerability func. midp.	SSP exposure
IO	2050	MIT	Vulnerability func. midp.	Vulnerability func. midp.	Vulnerability func. midp.	Vulnerability func. midp.
		CHAZ	Vulnerability func. midp.	Vulnerability func. midp.	Vulnerability func. midp.	Vulnerability func. midp.
		STORM	Vulnerability func. midp.	Vulnerability func. midp.	Vulnerability func. midp.	Vulnerability func. midp.
		IBTrACS_p	Vulnerability func. midp.	Vulnerability func. midp.	Vulnerability func. midp.	Vulnerability func. midp.
	2090	MIT	Vulnerability func. midp.	Vulnerability func. midp.	Vulnerability func. midp.	Vulnerability func. midp.
		CHAZ	Vulnerability func. midp.	Vulnerability func. midp.	Vulnerability func. midp.	Vulnerability func. midp.
		STORM	N/A	N/A	N/A	N/A
		IBTrACS_p	Vulnerability func. midp.	Vulnerability func. midp.	Vulnerability func. midp.	Vulnerability func. midp.
SH	2050	MIT	Vulnerability func. midp.	Vulnerability func. midp.	Vulnerability func. midp.	Vulnerability func. midp.
		CHAZ	Vulnerability func. midp.	Vulnerability func. midp.	Vulnerability func. midp.	Vulnerability func. midp.
		STORM	Vulnerability func. midp.	Vulnerability func. midp.	Vulnerability func. midp.	Vulnerability func. midp.
		IBTrACS_p	Vulnerability func. midp.	Vulnerability func. midp.	Vulnerability func. midp.	Vulnerability func. midp.
	2090	MIT	Vulnerability func. midp.	SSP exposure	Vulnerability func. midp.	SSP exposure
		CHAZ	Vulnerability func. midp.	Vulnerability func. midp.	Vulnerability func. midp.	Vulnerability func. midp.
		STORM	N/A	N/A	N/A	N/A
		IBTrACS_p	Vulnerability func. midp.	Vulnerability func. midp.	Vulnerability func. midp.	Vulnerability func. midp.
WP	2050	MIT	Vulnerability func. midp.	Vulnerability func. midp.	Vulnerability func. midp.	Vulnerability func. midp.
		CHAZ	Vulnerability func. midp.	Vulnerability func. midp.	Vulnerability func. midp.	Vulnerability func. midp.
		STORM	Vulnerability func. midp.	Vulnerability func. midp.	Vulnerability func. midp.	Vulnerability func. midp.
		IBTrACS_p	Vulnerability func. midp.	Vulnerability func. midp.	Vulnerability func. midp.	Vulnerability func. midp.
	2090	MIT	Vulnerability func. midp.	Vulnerability func. midp.	Vulnerability func. midp.	Vulnerability func. midp.
		CHAZ	Vulnerability func. midp.	Vulnerability func. midp.	Vulnerability func. midp.	Vulnerability func. midp.
		STORM	N/A	N/A	N/A	N/A
		IBTrACS_p	Vulnerability func. midp.	Vulnerability func. midp.	Vulnerability func. midp.	Vulnerability func. midp.

Table S3: Largest sensitivity indices for future TC risk estimates. Highest first- (S1) and total-order (ST) Sobol sensitivity indices for both risk metrics (expected annual damage (EAD) and 100-yr event (rp100)), expressed in absolute values (USD) in the four study regions (North Atlantic/Eastern Pacific (AP), North Indian Ocean (IO), Southern Hemisphere (SH), and North Western Pacific (WP) for both future periods (2050, 2090) and all four models (MIT, CHAZ, STORM, IBTrACS_p. Plots showing all sensitivity indices can be found in fig. S6 and fig. S7.

Institution	Model	Short name	Source
National Center for Atmospheric Research	CESM2	CESM2	Danabasoglu et al. (2020) (86)
Centre National de Recherches Météorologiques	CNRM-CM6-1	CNRM6	Voldoire et al. (2019) (87)
EC-Earth consortium	EC-Earth3	ECEARTH	EC-Earth Consortium (2019) (88)
Institute of Atmospheric Physics, Chinese Academy of Sciences	FGOALS-g3	FGOALS	Li et al. (2019) (89)
Institut Pierre Simon Laplace	IPSL-CM6A-LR	IPSL6	Hourdin et al. (2016) (90)
Japan Agency for Marine-Earth Science and Technology	MIROC6	MIROC6	Tatebe et al. (2019) (91)
Max Planck Institute	MPI-ESM1-2-HR	MPI2	Müller et al. (2018) (92)
Meteorological Research Institute, Tsukuba, Japan	MRI6-ESM2-0	MRI6	Yukimoto et al. (2019) (93)
United Kingdom Met Office	UKESM1-0-LL	UKMO6	Sellar et al. (2020) (94)

Table S4: List of CMIP6 models used in the downscaling of tropical cyclone event sets.

Model	TCR	TCR screen (likely)	ECS150	ECS130	ECS screen (likely)
CESM2	2.00	yes	5.15	6.43	no
CNRM-CM6-1	2.22	no	4.90	4.76	no
EC-Earth3	2.30	no	4.26	N/A	no
FGOALS-g3	1.50	yes	2.87	3.10	yes
IPSL-CM6A-LR	2.35	no	4.70	5.18	no
MIROC6	1.55	yes	2.60	2.59	yes
MPI-ESM1-2-HR	1.64	yes	2.98	3.34	yes
MRI6-ESM2-0	1.67	yes	3.13	3.42	yes
UKESM1-0-LL	2.77	no	5.36	5.49	no

Table S5: Transient climate response (TCR) and equilibrium climate sensitivity (ECS). TCR and ECS values for the nine GCMs, including a screen if the models fall into the likely range of projected TCR or ECS. Values are obtained from Hausfather et al. (2022) (95) supplementary data.

Other Supplementary Materials for this manuscript include the following:

Table S6: Statistical summary values of future tropical cyclone risk change drivers.
Statistical summary values of the boxplots shown in Fig. 2 (main text) and fig. S1 , fig. S2 , fig. S3 are provided in an extensive Excel table. The Excel file contains two sheets, one for each risk metric (EAD, rp100), which contain various statistical summary metrics for the different risk drivers (CC, SOC) and their product (sum; calculated form the sum of their log values) and total risk values (total) for the four hazard models (MIT, CHAZ, STORM, IBTrACS_p) in the four study regions (North Atlantic/Eastern Pacific (AP), North Indian Ocean (IO), Southern Hemisphere (SH), and North Western Pacific (WP)).

REFERENCES AND NOTES

1. J. M. Keenan, A climate intelligence arms race in financial markets. *Science* **365**, 1240–1243 (2019).

2. M Condon, Climate services: The business of physical risk (2023).

3. A. Arribas, R. Fairgrieve, T. Dhu, J. Bell, R. Cornforth, G. Gooley, C. J. Hilson, A. Luers, T. G. Shepherd, R. Street, N. Wood, Climate risk assessment needs urgent improvement. *Nat. Commun.* **13**, 4326 (2022).

4. T. Fiedler, A. J. Pitman, K. Mackenzie, N. Wood, C. Jakob, S. E. Perkins-Kirkpatrick, Business risk and the emergence of climate analytics. *Nat. Clim. Chang.* **11**, 87–94 (2021).

5. L. M. Braman, P. Suarez, M. K. van Aalst, Climate change adaptation: Integrating climate science into humanitarian work. *Intern. Rev. Red Cross* **92**, 693–712 (2010).

6. L. Jones, A. Dougill, R. G. Jones, A. Steynor, P. Watkiss, C. Kane, B. Koelle, W. Moufouma-Okia, J. Padgham, N. Ranger, J.-P. Roux, P. Suarez, T. Tanner, K. Vincent, Ensuring climate information guides long-term development. *Nat. Clim. Chang.* **5**, 812–814 (2015).

7. E. C. de Perez, S. J. Mason, Climate information for humanitarian agencies: Some basic principles. *Earth Perspect.* **1**, 11 (2014).

8. M. Enenkel, A. Kruczkiewicz, The humanitarian sector needs clear job profiles for climate science translators now more than ever. *Bull. Am. Meteorol. Soc.* **103**, E1088–E1097 (2022).

9. T. Geiger, K. Frieler, D. N. Bresch, A global historical data set of tropical cyclone exposure (TCE-DAT). *Earth Syst. Sci. Data* **10**, 185–194 (2018).

10. M. Berlemann, D. Wenzel, Hurricanes, economic growth and transmission channels: Empirical evidence for countries on differing levels of development. *World Dev.* **105**, 231–247 (2018).

11. R. Mendelsohn, K. Emanuel, S. Chonabayashi, L. Bakkensen, The impact of climate change on global tropical cyclone damage. *Nat. Clim. Chang.* **2**, 205–209 (2012).

12. A. Gettelman, D. N. Bresch, C. C. Chen, J. E. Truesdale, J. T. Bacmeister, Projections of future tropical cyclone damage with a high-resolution global climate model. *Clim. Change* **146**, 575–585 (2018).

13. C. M. Kropf, A. Ciullo, L. Otth, S. Meiler, A. Rana, E. Schmid, J. W. McCaughey, D. N. Bresch, Uncertainty and sensitivity analysis for probabilistic weather and climate-risk modelling: An implementation in CLIMADA v.3.1.0. *Geosci. Model Dev.* **15**, 7177–7201 (2022).

14. C.B. Field, V. Barros, T.F. Stocker, D. Qin, D.J. Dokken, K.L. Ebi, M.D. Mastrandrea, K. J. Mach, G.-K. Platner, S. K. Allen, M. Tignor, P. M. Midgley, IPCC, Managing the Risks of Extreme Events and Disasters to Advance Climate Change Adaptation. A Special Report of Working Groups I and II of the Intergovernmental Panel on Climate Change. (2012).

15. G. Aznar-Siguan, D. N. Bresch, CLIMADA v1: A global weather and climate risk assessment platform. *Geosci. Model Dev.* **12**, 3085–3097 (2019).

16. S. Meiler, T. Vogt, N. Bloemendaal, A. Ciullo, C.-Y. Lee, S. J. Camargo, K. Emanuel, D. N. Bresch, Intercomparison of regional loss estimates from global synthetic tropical cyclone models. *Nat. Commun.* **13**, 6156 (2022).

17. F. Pianosi, K. Beven, J. Freer, J. W. Hall, J. Rougier, D. B. Stephenson, T. Wagener, Sensitivity analysis of environmental models: A systematic review with practical workflow. *Environ. Model. Software* **79**, 214–232 (2016).

18. T. Wagener, R. Reinecke, F. Pianosi, On the evaluation of climate change impact models. *WIREs Clim. Chang.* **13**, e772 (2022).

19. W. Walker, P. Harremoës, J. Rotmans, J. van der Sluijs, M. van Asselt, P. Janssen, M. Krayer von Krauss, Defining uncertainty: A conceptual basis for uncertainty management in model-based decision support. *Integ. Assessment* **4**, 5–17 (2003).

20. E. Hawkins, R. Sutton, The potential to narrow uncertainty in regional climate predictions. *Bull. Am. Meteorol. Soc.* **90**, 1095–1108 (2009).

21. W. S. Parker, Predicting weather and climate: Uncertainty, ensembles and probability. *Stud. Hist. Philos. Sci. B Stud. Hist. Philos. Mod. Phys.* **41**, 263–272 (2010).

22. R. Knutti, Climate model confirmation: From philosophy to predicting climate in the real world in *Climate Modelling: Philosophical and Conceptual Issues*, E. A. Lloyd and E. Winsberg, Eds., (Springer International Publishing, 2018) 325–359.

23. R. H. Moss, J. A. Edmonds, K. A. Hibbard, M. R. Manning, S. K. Rose, D. P. van Vuuren, T. R. Carter, S. Emori, M. Kainuma, T. Kram, G. A. Meehl, J. F. B. Mitchell, N. Nakicenovic, K. Riahi, S. J. Smith, R. J. Stouffer, A. M. Thomson, J. P. Weyant, T. J. Wilbanks, The next generation of scenarios for climate change research and assessment. *Nature* **463**, 747–756 (2010).

24. R. Bradley, M. Drechsler, Types of uncertainty. *Erkenntnis* **79**, 1225–1248 (2014).

25. R. Bradley, K. Steele, Making climate decisions. *Philos. Compass* **10**, 799–810 (2015).

26. L. A. Mayer, K. Loa, B. Cwik, N. Tuana, K. Keller, C. Gonnerman, A. Parker, R. J. Lempert, Understanding scientists' computational modeling decisions about climate risk management strategies using values-informed mental models. *Glob. Environ. Chang.* **42**, 107–116 (2017).

27. S. O. Hansson, Evaluating the uncertainties. *The Argumentative Turn in Policy Analysis: Reasoning about Uncertainty*, S. O. Hansson, and G. Hirsch Hadorn, Eds., (Logic, Argumentation & Reasoning, Springer International Publishing, 2016) 79–104.

28. A. Saltelli, M. Ratto, T. Andres, F. Campolongo, J. Cariboni, D. Gatelli, M. Saisana, S. Tarantola, *Global Sensitivity Analysis: The Primer*. (John Wiley & Sons Ltd, 2008).

29. A. Saltelli, K. Aleksankina, W. Becker, P. Fennell, F. Ferretti, N. Holst, S. Li, Q. Wu, Why so many published sensitivity analyses are false: A systematic review of sensitivity analysis practices. *Environ. Model. Softw.* **114**, 29–39 (2019).

30. S. Meiler, A. Ciullo, C. M. Kropf, K. Emanuel, D. N. Bresch, Uncertainties and sensitivities in the quantification of future tropical cyclone risk. *Commun. Earth Environ.* **4**, 371 (2023).

31. L. C. Dawkins, D. J. Bernie, F. Pianosi, J. A. Lowe, T. Economou, Quantifying uncertainty and sensitivity in climate risk assessments: Varying hazard, exposure and vulnerability modelling choices. *Clim. Risk Manag.* **40**, 100511 (2023).

32. S. Meiler, A. Ciullo, D. N. Bresch, C. M. Kropf, Uncertainty and sensitivity analysis for probabilistic, global modelling of future tropical cyclone risk, *14th International Conference on Applications of Statistics and Probability in Civil Engineering (ICASP14)* (2023). https://doi.org/10.25546/103244.

33. S. Lo Piano, R. Sheikholeslami, A. Puy, A. Saltelli, Unpacking the modelling process via sensitivity auditing. *Futures* **144**, 103041 (2022).

34. T. Page, P. Smith, K. Beven, F. Pianosi, F. Sarrazin, S. Almeida, L. Holcombe, J. Freer, N. Chappell, T. Wagener, Technical note: The CREDIBLE Uncertainty Estimation (CURE) toolbox: Facilitating the communication of epistemic uncertainty. *Hydrol. Earth Syst. Sci.* **27**, 2523–2534 (2023).

35. K. Emanuel, S. Ravela, E. Vivant, C. Risi, A statistical deterministic approach to hurricane risk assessment. *Bull. Am. Meteorol. Soc.* **87**, 299–314 (2006).

36. K. Emanuel, The hurricane—Climate connection. *Bull. Am. Meteorol. Soc.* **89**, ES10–ES20 (2008).

37. C.-Y. Lee, M. K. Tippett, A. H. Sobel, S. J. Camargo, An environmentally forced tropical cyclone hazard model. *J. Adv. Model. Earth Syst.* **10**, 223–241 (2018).

38. C.-Y. Lee, S. J. Camargo, A. H. Sobel, M. K. Tippett, Statistical–dynamical downscaling projections of tropical cyclone activity in a warming climate: Two diverging genesis scenarios. *J. Climate* **33**, 4815–4834 (2020).

39. N. Bloemendaal, I. D. Haigh, H. de Moel, S. Muis, R. J. Haarsma, J. C. J. H. Aerts, Generation of a global synthetic tropical cyclone hazard dataset using STORM. *Sci. Data* **7**, 40 (2020).

40. N. Bloemendaal, H. de Moel, A. B. Martinez, S. Muis, I. D. Haigh, K. van der Wiel, R. J. Haarsma, P. J. Ward, M. J. Roberts, J. C. M. Dullaart, J. C. J. H. Aerts, A globally consistent local-scale assessment of future tropical cyclone risk. *Sci. Adv.* **8**, eabm8438 (2022).

41. S. Kleppek, V. Muccione, C. C. Raible, D. N. Bresch, P. Koellner-Heck, T. F. Stocker, Tropical cyclones in ERA-40: A detection and tracking method. *Geophys. Res. Lett.* **35**, L10705 (2008).

42. K. R. Knapp, M. C. Kruk, D. H. Levinson, H. J. Diamond, C. J. Neumann, The international best track archive for climate stewardship (IBTrACS). *Bull. Am. Meteorol. Soc.* **91**, 363–376 (2010).

43. K. Riahi, D. P. van Vuuren, E. Kriegler, J. Edmonds, B. C. O'Neill, S. Fujimori, N. Bauer, K. Calvin, R. Dellink, O. Fricko, W. Lutz, A. Popp, J. C. Cuaresma, S. Kc, M. Leimbach, L. Jiang, T. Kram, S. Rao, J. Emmerling, K. Ebi, T. Hasegawa, P. Havlik, F. Humpenöder, L. A. Da Silva, S. Smith, E. Stehfest, V. Bosetti, J. Eom, D. Gernaat, T. Masui, J. Rogelj, J. Strefler, L. Drouet, V. Krey, G. Luderer, M. Harmsen, K. Takahashi, L. Baumstark, J. C. Doelman, M. Kainuma, Z. Klimont, G. Marangoni, H. Lotze-Campen, M. Obersteiner, A. Tabeau, M. Tavoni, The shared socioeconomic pathways and their energy, land use, and greenhouse gas emissions implications: An overview. *Glob. Environ. Chang.* **42**, 153–168 (2017).

44. R. Dellink, J. Chateau, E. Lanzi, B. Magné, Long-term economic growth projections in the shared socioeconomic pathways. *Glob. Environ. Chang.* **42**, 200–214 (2017).

45. J. Crespo Cuaresma, Income projections for climate change research: A framework based on human capital dynamics. *Glob. Environ. Chang.* **42**, 226–236 (2017).

46. M. Leimbach, E. Kriegler, N. Roming, J. Schwanitz, Future growth patterns of world regions – A GDP scenario approach. *Glob. Environ. Chang.* **42**, 215–225 (2017).

47. S. Eberenz, S. Lüthi, D. N. Bresch, Regional tropical cyclone impact functions for globally consistent risk assessments. *Nat. Hazard s Earth Syst. Sci.* **21**, 393–415 (2021).

48. K. A. Emanuel, Global warming effects on U.S. hurricane damage. *Weather Clim. Soc.* **3**, 261–268 (2011).

49. K. M. Wilson, J. W. Baldwin, R. M. Young, Estimating tropical cyclone vulnerability: A review of different open-source approaches, in *Hurricane Risk in a Changing Climate*, J. M. Collins, J. M. Done, Eds. (Hurricane Risk, Springer International Publishing, 2022), pp. 255–281.

50. C. Lemieux, *Monte Carlo and Quasi-Monte Carlo Sampling* (Springer Science & Business Media, 2009).

51. C. Unterberger, P. Hudson, W. J. Botzen, K. Schroeer, K. W. Steininger, Future public sector flood risk and risk sharing arrangements: An assessment for Austria. *Ecol. Econ.* **156**, 153–163 (2019).

52. T. Knutson, S. J. Camargo, J. C. Chan, K. Emanuel, C. H. Ho, J. Kossin, M. Mohapatra, M. Satoh, M. Sugi, K. Walsh, L. Wu, Tropical cyclones and climate change assessment part II: Projected response to anthropogenic warming. *Bull. Am. Meteorol. Soc.* **101**, E303–E322 (2020).

53. I. M. Sobol, Global sensitivity indices for nonlinear mathematical models and their Monte Carlo estimates. *Math. Comput. Simul.* **55**, 271–280 (2001).

54. A. Saltelli, P. Annoni, I. Azzini, F. Campolongo, M. Ratto, S. Tarantola, Variance based sensitivity analysis of model output. Design and estimator for the total sensitivity index. *Comput. Phys. Commun.* **181**, 259–270 (2010).

55. K. Emanuel, Environmental factors affecting tropical cyclone power dissipation. *J. Climate* **20**, 5497–5509 (2007).

56. K. A. Emanuel, D. S. Nolan, Tropical cyclone activity and global climate, *26th Conference on Hurricanes and Tropical Meteorology* (Amer. Meteor. Soc., 2004), pp. 240–241.

57. K. Emanuel, Tropical cyclone activity downscaled from NOAA-CIRES reanalysis, 1908–1958. *J. Adv. Model. Earth Syst.* **2**, 1 (2010).

58. E. D. Rappin, D. S. Nolan, K. A. Emanuel, Thermodynamic control of tropical cyclogenesis in environments of radiative-convective equilibrium with shear: Tropical cyclogenesis in variable climates. *Q. J. Roy. Meteorol. Soc.* **136**, 1954–1971 (2010).

59. A. H. Sobel, A. A. Wing, S. J. Camargo, C. M. Patricola, G. A. Vecchi, C.-Y. Lee, M. K. Tippett, Tropical cyclone frequency. *Earth's Future* **9**, e2021EF002275 (2021).

60. A. Puy, P. Beneventano, S. A. Levin, S. Lo Piano, T. Portaluri, A. Saltelli, Models with higher effective dimensions tend to produce more uncertain estimates. *Sci. Adv.* **8**, eabn9450 (2022).

61. J. Roussos, R. Bradley, R. Frigg, Making confident decisions with model ensembles. *Philos. Sci.* **88**, 439–460 (2021).

62. M. Golnaraghi, P. Nunn, R. Muir-Wood, J. Guin, D. Whitaker, J. Slingo, G. Asrar, I. Branagan, G. Lemcke, C. Souch, M. Jean, A. Allman, M. Jahn, D. Bresch, P. Khalil, M. Beck, Managing Physical Risks of Climate: Leveraging Innovations in Catastrophe Risk Modelling: Reseach Brief, (November), 2018 (2018); https://api.semanticscholar.org/CorpusID:134139721.

63. A. Ciullo, E. Strobl, S. Meiler, O. Martius, D. Bresch, Increasing countries' financial resilience through global catastrophe risk pooling. *Nat. Commun.* **14**, 922 (2023).

64. J. A. Curry, P. J. Webster, Climate science and the uncertainty monster. *Bull. Am. Meteorol. Soc.* **92**, 1667–1682 (2011).

65. T. Geiger, J. Gütschow, D. N. Bresch, K. Emanuel, K. Frieler, Double benefit of limiting global warming for tropical cyclone exposure. *Nat. Clim. Change* **11**, 861–866 (2021).

66. K. J. Beven, S. Almeida, W. P. Aspinall, P. D. Bates, S. Blazkova, E. Borgomeo, J. Freer, K. Goda, J. W. Hall, J. C. Phillips, M. Simpson, P. J. Smith, D. B. Stephenson, T. Wagener, M. Watson, K. L. Wilkins, Epistemic uncertainties and natural hazard risk assessment – Part 1: A review of different natural hazard areas. *Nat. Hazards Earth Syst. Sci.* **18**, 2741–2768 (2018).

67. M. Henrion, M. G. Morgan, The nature and sources of uncertainty, in *Uncertainty: A Guide to Dealing with Uncertainty in Quantitative Risk and Policy Analysis* (Cambridge Univ. Press, 1990), pp. 47–72.

68. J. Gao, Global 1-km Downscaled Population Base Year and Projection Grids Based on the Shared Socioeconomic Pathways, Revision 01. NASA Socioeconomic Data and Applications Center (SEDAC), Palisades, New York (2020); https://doi.org/10.7927/q7z9-9r69; https://www.earthdata.nasa.gov/centers/sedac-daac

69. T. Wang, F. Sun, Global gridded GDP data set consistent with the shared socioeconomic pathways. *Sci. Data* **9**, 221 (2022).

70. G. Aznar-Siguan, E. Schmid, T. Vogt, S. Eberenz, C. B. Steinmann, T. Röösli, Y. Yu, E. Mühlhofer, S. Lüthi, I. J. Sauer, J. Hartman, C. M. Kropf, B. P. Guillod, Z. Stalhandske, A. Ciullo, D. N. Bresch, L. Riedel, C. Fairless, T. Schmid, P. M. M. Kam, N. Colombi, wjan262, S. Meiler, leonie-villiger, climada-jenkins, Rachel_B, raphael-portmann, veronicabozzini, DarioStocker, and scem, CLIMADA-project/climada_python: v4.0.1. Zenodo (2023); https://doi.org/10.5281/zenodo.8383171, doi:10.5281/zenodo.8383171, 13, 340, 348, 2019.

71. G. Holland, A revised hurricane pressure-wind model. *Mon. Weather Rev.* **136**, 3432–3445 (2008).

72. K. Emanuel, R. Rotunno, Self-stratification of tropical cyclone outflow. Part I: Implications for storm structure. *J. Atmos. Sci.* **68**, 2236–2249 (2011).

73. D. G. Marks, The Beta and advection model for hurricane track forecasting (1992); https://repository.library.noaa.gov/view/noaa/7184.

74. H. Hersbach, B. Bell, P. Berrisford, A. Horányi, J. M. Sabater, J. Nicolas, R. Radu, D. Schepers, A. Simmons, C. Soci, D. Dee, Global reanalysis: Goodbye ERA-Interim, hello ERA5. *ECMWF Newsletter* **159**, 17–24 (2019).

75. T. R. Knutson, J. J. Sirutis, M. Zhao, R. E. Tuleya, M. Bender, G. A. Vecchi, G. Villarini, D. Chavas, Global projections of intense tropical cyclone activity for the late twenty-first

century from dynamical downscaling of CMIP5/RCP4.5 scenarios. *J. Climate* **28**, 7203–7224 (2015).

76. A. H. Sobel, C. Y. Lee, S. J. Camargo, K. T. Mandli, K. A. Emanuel, P. Mukhopadhyay, M. Mahakur, Tropical cyclone hazard to mumbai in the recent historical climate. *Mon. Weather Rev.* **147**, 2355–2366 (2019).

77. IIASA, RCP Database (Version 2.0.5) (2009); https://tntcat.iiasa.ac.at/RcpDb/, Accessed on 2023-09-18.

78. S. Eberenz, D. Stocker, T. Röösli, D. N. Bresch, Asset exposure data for global physical risk assessment. *Earth Syst. Sci. Data* **12**, 817–833 (2020).

79. J. Herman, W. Usher, SALib: An open-source python library for sensitivity analysis. *J. Open Source Softw.* **2**, 97 (2017).

80. N. Bloemendaal, I. I. Haigh, H. H. de Moel, S. Muis, R. R. Haarsma, J. J. Aerts, STORM IBTrACS present climate synthetic tropical cyclone tracks. 4TU.Centre for Research Data (2020); https://data.4tu.nl/articles/_/12706085/2, Accessed on 2024-02-29.

81. N. Bloemendaal, H. H. de Moel, A. B. Martinez, S. S. Muis, I. I. Haigh, K. van der Wiel, R. R. Haarsma, P. P. Ward, M. Roberts, J. Dullaart, STORM Climate Change synthetic tropical cyclone tracks. 4TU.ResearchData (2023); https://data.4tu.nl/datasets/98900e17-8e01-4d70-b3b6-ca1a1da2f194/2, Accessed on 2024-02-29.

82. S. Meiler, simonameiler/TC_future_risk_uncertainty_multi-model (2024); https://github.com/simonameiler/TC_future_risk_uncertainty_multi-model, Accessed on 2024-02-29.

83. M. K. Tippett, S. J. Camargo, A. H. Sobel, A poisson regression index for tropical cyclone genesis and the role of large-scale vorticity in genesis. *J. Climate* **24**, 2335–2357 (2011).

84. S. J. Camargo, M. K. Tippett, A. H. Sobel, G. A. Vecchi, M. Zhao, Testing the performance of tropical cyclone genesis indices in future climates using the HiRAM model. *J. Climate* **27**, 9171–9196 (2014).

85. K. Emanuel, Response of global tropical cyclone activity to increasing CO_2: Results from downscaling CMIP6 models. *J. Climate* **34**, 57–70 (2021).

86. G. Danabasoglu, J.-F. Lamarque, J. Bacmeister, D. A. Bailey, A. K. DuVivier, J. Edwards, L. K. Emmons, J. Fasullo, R. Garcia, A. Gettelman, C. Hannay, M. M. Holland, W. G. Large, P. H. Lauritzen, D. M. Lawrence, J. T. M. Lenaerts, K. Lindsay, W. H. Lipscomb, M. J. Mills, R. Neale, K. W. Oleson, B. Otto-Bliesner, A. S. Phillips, W. Sacks, S. Tilmes, L. van Kampenhout, M. Vertenstein, A. Bertini, J. Dennis, C. Deser, C. Fischer, B. Fox-Kemper, J. E. Kay, D. Kinnison, P. J. Kushner, V. E. Larson, M. C. Long, S. Mickelson, J. K. Moore, E. Nienhouse, L. Polvani, P. J. Rasch, W. G. Strand, The community Earth system model version 2 (CESM2). *J. Adv. Model. Earth Syst.* **12**, e2019MS001916 (2020).

87. A. Voldoire, D. Saint-Martin, S. Sénési, B. Decharme, A. Alias, M. Chevallier, J. Colin, J.-F. Guérémy, M. Michou, M.-P. Moine, P. Nabat, R. Roehrig, D. Salas y Mélia, D. S. y Mélia, R. Séférian, S. Valcke, I. Beau, S. Belamari, S. Berthet, C. Cassou, J. Cattiaux, J. Deshayes, H. Douville, C. Ethé, L. Franchistéguy, O. Geoffroy, C. Lévy, G. Madec, Y. Meurdesoif, R. Msadek, A. Ribes, E. Sanchez-Gomez, L. Terray, R. Waldman, Evaluation of CMIP6 DECK experiments with CNRM-CM6-1. *J. Adv. Model. Earth Syst.* **11**, 2177–2213 (2019).

88. EC Earth Consortium, EC-Earth-Consortium EC-Earth3 model output prepared for CMIP6 ScenarioMIP ssp245 (2019); https://doi.org/10.22033/ESGF/CMIP6.4880, Accessed on 2023-02-16.

89. L. Li, CAS FGOALS-g3 model output prepared for CMIP6 ScenarioMIP ssp245 (2019); https://doi.org/10.22033/ESGF/CMIP6.3469, Accessed on 2023-02-16.

90. F. Hourdin, C. Rio, J.-Y. Grandpeix, J.-B. Madeleine, F. Cheruy, N. Rochetin, A. Jam, I. Musat, A. Idelkadi, L. Fairhead, M.-A. Foujols, L. Mellul, A.-K. Traore, J.-L. Dufresne, O. Boucher, M.-P. Lefebvre, E. Millour, E. Vignon, J. Jouhaud, F. B. Diallo, F. Lott, G. Gastineau, A. Caubel, Y. Meurdesoif, J. Ghattas, LMDZ6A: The atmospheric component of the IPSL climate model with improved and better tuned physics. *J. Adv. Model. Earth Syst.* **12**, e2019MS001892 (2020).

91. H. Tatebe, T. Ogura, T. Nitta, Y. Komuro, K. Ogochi, T. Takemura, K. Sudo, M. Sekiguchi, M. Abe, F. Saito, M. Chikira, S. Watanabe, M. Mori, N. Hirota, Y. Kawatani, T. Mochizuki, K. Yoshimura, K. Takata, R. O'ishi, D. Yamazaki, T. Suzuki, M. Kurogi, T. Kataoka, M. Watanabe, M. Kimoto, Description and basic evaluation of simulated mean state, internal variability, and climate sensitivity in MIROC6. *Geosci. Model Dev.* **12**, 2727–2765 (2019).

92. W. A. Müller, J. H. Jungclaus, T. Mauritsen, J. Baehr, M. Bittner, R. Budich, F. Bunzel, M. Esch, R. Ghosh, H. Haak, T. Ilyina, T. Kleine, L. Kornblueh, H. Li, K. Modali, D. Notz, H. Pohlmann, E. Roeckner, I. Stemmler, F. Tian, J. Marotzke, A higher-resolution version of the Max Planck Institute Earth System Model (MPI-ESM1.2-HR). *J. Adv. Model. Earth Syst.* **10**, 1383–1413 (2018).

93. S. Yukimoto, T. Koshiro, H. Kawai, N. Oshima, K. Yoshida, S. Urakawa, H. Tsujino, M. Deushi, T. Tanaka, M. Hosaka, H. Yoshimura, E. Shindo, R. Mizuta, M. Ishii, A. Obata, Y. Adachi, MRI MRI-ESM2.0 model output prepared for CMIP6 ScenarioMIP ssp245 (2019); https://doi.org/10.22033/ESGF/CMIP6.6910, Accessed on 2023-02-16.

94. A. A. Sellar, J. Walton, C. G. Jones, R. Wood, N. L. Abraham, M. Andrejczuk, M. B. Andrews, T. Andrews, A. T. Archibald, L. de Mora, H. Dyson, M. Elkington, R. Ellis, P. Florek, P. Good, L. Gohar, S. Haddad, S. C. Hardiman, E. Hogan, A. Iwi, C. D. Jones, B. Johnson, D. I. Kelley, J. Kettleborough, J. R. Knight, M. O. Köhler, T. Kuhlbrodt, S. Liddicoat, I. Linova-Pavlova, M. S. Mizielinski, O. Morgenstern, J. Mulcahy, E. Neininger, F. M. O'Connor, R. Petrie, J. Ridley, J.-C. Rioual, M. Roberts, E. Robertson, S. Rumbold, J. Seddon, H. Shepherd, S. Shim, A. Stephens, J. C. Teixiera, Y. Tang, J. Williams, A. Wiltshire, P. T. Griffiths, Implementation of U.K. Earth system models for CMIP6. *J. Adv. Model. Earth Syst.* **12**, e2019MS001946 (2020).

95. Z. Hausfather, K. Marvel, G. A. Schmidt, J. W. Nielsen-Gammon, M. Zelinka, Climate simulations: Recognize the 'hot model' problem. *Nature* **605**, 26–29 (2022).
